# The Maintenance of Synaptic Homeostasis at the *Drosophila* Neuromuscular Junction Is Reversible and Sensitive to High Temperature

**DOI:** 10.1523/ENEURO.0220-17.2017

**Published:** 2017-12-12

**Authors:** Catherine J. Yeates, Danielle J. Zwiefelhofer, C. Andrew Frank

**Affiliations:** 1 Department of Anatomy and Cell Biology, University of Iowa Carver College of Medicine, Iowa City, IA 52242; 2 Interdisciplinary Graduate Program in Neuroscience, University of Iowa, Iowa City, IA 52242; 3 Interdisciplinary Programs in Neuroscience, Genetics, and Molecular Medicine, University of Iowa, Iowa City, IA 52242

**Keywords:** Drosophila, homeostatic, neuromuscular, plasticity, reversibility, synapse

## Abstract

Homeostasis is a vital mode of biological self-regulation. The hallmarks of homeostasis for any biological system are a baseline set point of physiological activity, detection of unacceptable deviations from the set point, and effective corrective measures to counteract deviations. Homeostatic synaptic plasticity (HSP) is a form of neuroplasticity in which neurons and circuits resist environmental perturbations and stabilize levels of activity. One assumption is that if a perturbation triggers homeostatic corrective changes in neuronal properties, those corrective measures should be reversed upon removal of the perturbation. We test the reversibility and limits of HSP at the well-studied *Drosophila melanogaster* neuromuscular junction (NMJ). At the *Drosophila* NMJ, impairment of glutamate receptors causes a decrease in quantal size, which is offset by a corrective, homeostatic increase in the number of vesicles released per evoked presynaptic stimulus, or quantal content. This process has been termed presynaptic homeostatic potentiation (PHP). Taking advantage of the GAL4/GAL80^TS^/UAS expression system, we triggered PHP by expressing a dominant-negative glutamate receptor subunit at the NMJ. We then reversed PHP by halting expression of the dominant-negative receptor. Our data show that PHP is fully reversible over a time course of 48–72 h after the dominant-negative glutamate receptor stops being genetically expressed. As an extension of these experiments, we find that when glutamate receptors are impaired, neither PHP nor NMJ growth is reliably sustained at high culturing temperatures (30–32°C). These data suggest that a limitation of homeostatic signaling at high temperatures could stem from the synapse facing a combination of challenges simultaneously.

## Significance Statement

Biological homeostatic systems must upregulate or downregulate cellular parameters to maintain appropriate set points of physiologic activity. Homeostasis is a well-documented mode of regulation in metazoan nervous systems. True homeostatic control should be a reversible process. Because of technical difficulties of presenting and removing functional challenges to living synapses, the reversibility of homeostatic forms of synapse regulation has not been rigorously examined *in vivo* over extended developmental time. Here we formally demonstrate that a form of long-term homeostatic regulation of *Drosophila melanogaster* neuromuscular synapse function is reversible and temperature labile. This is significant because developing methods to study how homeostatic regulatory systems are turned on and off could lead to fundamental new insights about control of synaptic output.

## Introduction

Homeostasis is a strong form of biological regulation. It permits individual cells or entire systems of cells to maintain core physiologic properties that are compatible with life. In the nervous system, decades of study have shown that while synapses and circuits are inherently plastic, they also possess robust homeostatic regulatory systems to maintain physiologic stability. Homeostatic plasticity in the nervous system is a non-Hebbian strategy to counteract challenges to neuronal function that may threaten to disrupt essential neuronal and circuit activities (Turrigiano, 2017). Depending on the synaptic preparation examined and the environmental challenge presented to the synapse, homeostatic responses may be executed via compensatory adjustments to presynaptic neurotransmitter release (Cull-Candy et al. 1980; Petersen et al. 1997; Murthy et al. 2001; Thiagarajan et al. 2005; Frank et al. 2006; Davis and Müller, 2015), postsynaptic neurotransmitter receptor composition (O’Brien et al. 1998; Turrigiano et al. 1998; Rongo and Kaplan, 1999; Turrigiano, 2008), neuronal excitability (Marder and Prinz, 2002; Marder and Goaillard, 2006; Marder and Bucher, 2007; Bergquist et al. 2010; Parrish et al. 2014), or even developmentally, via changes in synaptic contact formation and maintenance (Davis and Goodman, 1998; Burrone et al. 2002; Wefelmeyer et al. 2016).

Bidirectionality has been documented in several homeostatic systems, perhaps most prominently in the case of synaptic scaling of neurotransmitter receptors. For vertebrate neuronal culture preparations—such as cortical neurons or spinal neurons—global silencing of network firing can induce increases in excitatory properties, such as increased AMPA-type glutamate receptor accumulation; by contrast, global enhancement of activity can induce the opposite type of response (O’Brien et al. 1998; Turrigiano et al. 1998; Wierenga et al. 2005; Turrigiano, 2008). Bidirectionality is also a key feature underlying homeostatic alterations of neurotransmitter release at peripheral synapses such as the neuromuscular junction (NMJ). At the NMJs of *Drosophila melanogaster* and mammals, impairing neurotransmitter receptor function postsynaptically results in diminished sensitivity to single vesicles of transmitter. Electrophysiologically, this manifests as decreased quantal size. NMJs respond to this challenge by enhancing neurotransmitter vesicle release (Cull-Candy et al. 1980; Plomp et al. 1992, 1995; Petersen et al. 1997; Davis et al. 1998; Frank et al. 2009). By contrast, perturbations that enhance quantal size—for example, overexpression of a vesicular neurotransmitter transporter in *Drosophila*—can result in decreased quantal content (Daniels et al. 2004; Gaviño et al. 2015).

Synapses and circuits possess myriad solutions to assume appropriate functional outputs in the face of perturbations (Marder and Prinz, 2002; Marder and Goaillard, 2006). Therefore, a corollary to bidirectional regulation is that homeostatic forms of regulation should also be reversible. There are experimental difficulties of presenting and removing a synaptic challenge in the context of a living synapse, so homeostatic reversibility has not been rigorously studied in an *in vivo* system or over extended periods of developmental time. Understanding how homeostatic regulatory systems are reversibly turned on and off could have profound implications for elucidating fundamental properties of circuit regulation.

Here we exploit the *Drosophila* NMJ as a living synapse to test homeostatic reversibility. At the *Drosophila* NMJ, a canonical way to challenge synapse function is through glutamate receptor impairment (Frank, 2014), either genetically (Petersen et al. 1997) or pharmacologically (Frank et al. 2006). Impairments of muscle glutamate receptor function decrease quantal size. Decreased quantal size spurs muscle-to-nerve signaling that ultimately results in a homeostatic increase in presynaptic vesicle release, a process that has been termed presynaptic homeostatic potentiation (PHP). The most widely used experimental homeostatic challenges to *Drosophila* NMJ function are not easily reversed. These challenges include genetic deletion of the glutamate receptor subunit GluRIIA (Petersen et al. 1997) and the mostly irreversible pharmacological inhibition of glutamate receptors with Philanthotoxin-433 (PhTox; Frank et al. 2006).

For this study, we engineered a way to challenge NMJ function *in vivo* for significant periods of time, verify the effectiveness of the challenge at a defined developmental time point, remove the challenge, and then assess the homeostatic capacity of the NMJ at a later developmental time point. By using the temporal and regional gene expression targeting (TARGET) GAL4/GAL80^TS^/UAS expression system (McGuire et al. 2003) to temporally control the expression of a dominant-negative GluRIIA receptor subunit (DiAntonio et al. 1999), we found that homeostatic potentiation of neurotransmitter release is fully reversible. In the course of conducting our studies, we also uncovered a high temperature limitation of homeostatic potentiation at the NMJ.

## Materials and Methods

### *Drosophila* stocks and husbandry

Fruit fly stocks were obtained from either the Bloomington Drosophila Stock Center (BDSC) or the labs that generated them. *w^1118^* was used as a wild-type (WT) control (Hazelrigg et al. 1984). The *GluRIIA^SP16^* deletion was used as a genetic loss-of-function (Petersen et al. 1997). Transgenes included the *UAS-*driven dominant-negative glutamate receptor subunit, *UAS-GluRIIA^M614R^*(DiAntonio et al. 1999), and a ubiquitous *Tubulin_Promoter_-Gal80^TS^*(*Tub_P_-Gal80^TS^*; McGuire et al. 2003). Muscle-specific GAL4 drivers included *MHC-Gal4* (Schuster et al. 1996a, b) and *BG57-Gal4* (also commonly known as *C57-Gal4*; Budnik et al. 1996). For reversibility experiments, the full genotypes for the crosses were *w^1118^; CyO-GFP/UAS-GluRIIA^M614R^; TM6b(Tb)/Tub_P_-Gal80^TS^* males × *w^1118^;; TM6b(Tb)/MHC-Gal4 or BG57-Gal4* virgin females. Non-tubby, non-GFP larvae were selected for recording. In control recordings, we found no discernable differences between male and female third-instar electrophysiology, but for reversibility experiments (Figs. 4–6), single sexes of larvae were chosen to eliminate sex as a possible confounding variable.

Fruit flies were raised on cornmeal, molasses, and yeast medium (see BDSC website for standard recipe) in temperature-controlled conditions. For most experiments, animals were reared at the temperatures noted (including temperature shifts) until they reached the wandering third instar larval stage, at which point they were chosen for electrophysiological recording. For experiments in Figs. 3 and 4, mated animals were placed at either 21°C, 25°C, or 29°C and allowed to lay eggs for 6–8 h. Stage- and size-matched early third instar larvae (∼48–54 h after egg laying) were subjected to electrophysiological recording (Fig. 3) or temperature swaps (Figs. 4–6), as indicated.

### Electrophysiology and analysis

**Figure 1.**
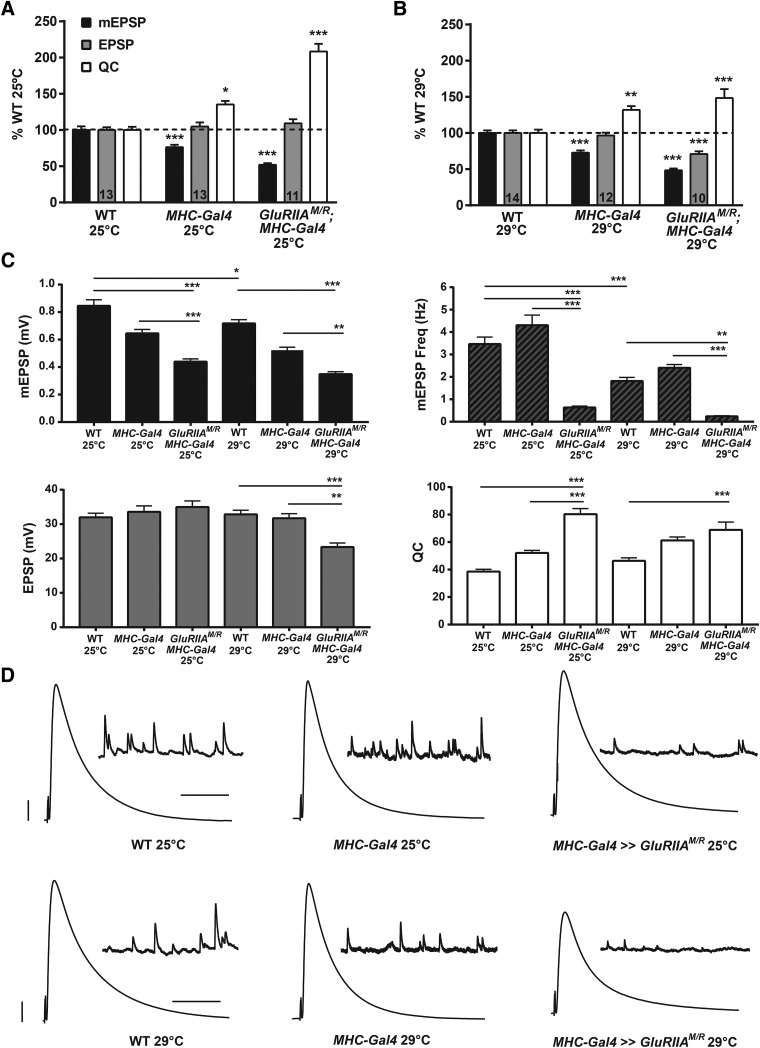
Postsynaptic expression of GluRIIA^M/R^ causes a decrease in mEPSP size and a homeostatic increase in quantal content. ***A***, Electrophysiological profiles comparing WT (*w^1118^*) NMJs to NMJs expressing driver alone (*w;; MHC-Gal4/+*) and NMJs with postsynaptic expression of a dominant-negative transgene *UAS-GluRIIA^M/R^* (*w; UAS-GluRIIA^M/R^/+; MHC-Gal4/+*). Animals were reared at 25°C. Driver controls showed a significant decrease in mEPSP amplitude (***p < 0.001) and increase in quantal content (*p < 0.05) compared to WT. mEPSP amplitude in the dominant-negative was even more markedly decreased compared to WT (***p < 0.001). Quantal content (QC) was significantly increased for the dominant-negative NMJs (***p < 0.001), revealing a homeostatic system that maintains EPSP amplitude at control levels. ***B***, The same genotypes as in ***A***, raised at 29°C. Driver controls showed a baseline decrease in mEPSP amplitude (***p < 0.001) and an increase in quantal content (**p < 0.01). mEPSP amplitude was significantly decreased in the dominant-negative (***p < 0.001) compared to WT. EPSP amplitude was also decreased in the dominant-negative compared to WT (***p < 0.001), with QC significantly increased (***p < 0.001) but not enough to completely offset the decrease in quantal size. ***C***, Raw value comparisons of the recordings normalized in ***A*** and ***B***. mEPSP amplitude, mEPSP frequency, EPSP amplitude, and QC were compared for all six conditions. In addition to the observations above, WT at 29°C had decreased mEPSP amplitude (*p < 0.05) and mEPSP frequency (***p < 0.001) compared to WT at 25°C. There was no difference in quantal content between the two WT conditions (*p* = 0.38). mEPSP amplitude was decreased in the dominant-negatives compared to driver controls at 25°C (***p < 0.001) and 29°C (**p < 0.01). At 25°C, the dominant-negative showed a marked decrease in mEPSP frequency compared to WT at the same temperature (***p < 0.001). The dominant-negative at 29°C also showed a significant decrease in mEPSP frequency compared to WT at 29°C (**p < 0.01). Dominant-negative animals also showed significant decreases in mEPSP frequency compared to driver controls at their respective temperatures (***p < 0.001). QC was significantly increased in dominant-negative animals compared to driver controls at 25°C (***p < 0.001). ***D***, Electrophysiological traces. Scale bars for EPSPs (and mEPSPs) are 5 mV (1 mV) and 50 ms (1000 ms). All statistical comparisons done by one-way ANOVA with Tukey’s *post hoc*, collectively comparing the six total conditions.

**Figure 2.**
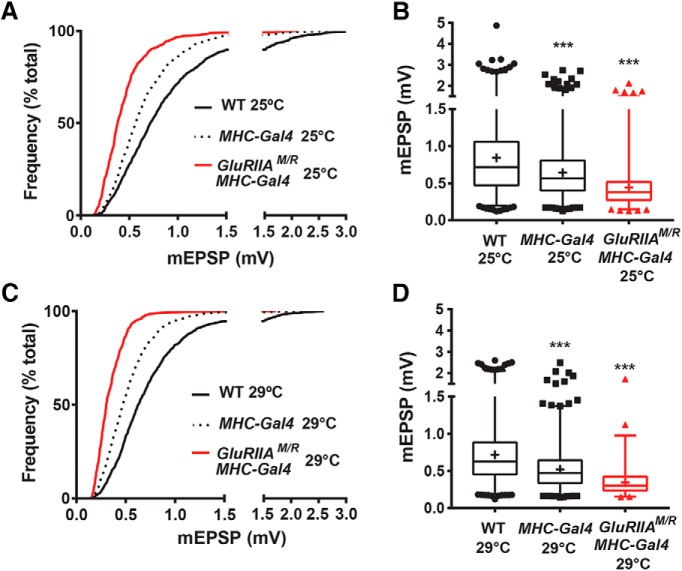
mEPSP amplitude distribution is leftward-shifted for muscle GAL4 driver controls and dominant-negative NMJs. ***A***, Cumulative frequency distributions plotting mEPSP values for WT, *w;; MHC-Gal4/+*, and *w; UAS-GluRIIA^M/R^/+; MHC-GAL4/+* NMJs at 25°C. ***B***, Box-and-whisker plots of mEPSP amplitude for the same genotypes. Driver controls and dominant-negatives both showed significantly diminished mEPSP distribution profiles compared to WT (***p < 0.001, Kruskal-Wallis test). ***C***, Cumulative frequency distribution comparing mEPSP values for WT, *w;; MHC-Gal4/+*, and *w; UAS-GluRIIA^M/R^/+; MHC-GAL4/+* at 29°C. ***D***, Box-and-whisker plots of mEPSP amplitude for the same genotypes. Driver controls and dominant-negatives both show significantly diminished mEPSP distribution profiles compared to WT (***p < 0.001, Kruskal-Wallis test).

**Figure 3.**
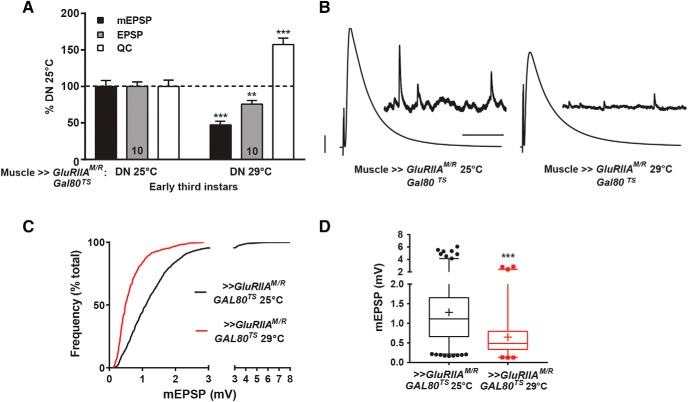
Animals expressing GluRIIA^M/R^ show homeostatic compensation early in development. ***A***, *w; GluRIIA^M/R^/+; MHC-GAL4/GAL80^TS^* animals were reared at 25°C or 29°C, and recordings were performed on early third instar larvae. The animals reared at 29°C had a marked decrease in average mEPSP amplitude compared to animals reared at 25°C (***p < 0.001, Student’s *t* test), as well as a decrease in mEPSP frequency (*p* < 0.001, see Table 1). EPSP amplitude was decreased at 29°C (**p < 0.01), but there was a significant increase in QC at 29°C (***p < 0.001). ***B***, Representative electrophysiological traces. Scale bars for EPSPs (and mEPSPs) are 5 mV (1 mV) and 50 ms (1000 ms). ***C***, Cumulative frequency distribution comparing mEPSP values for the 25°C versus 29°C conditions. ***D***, Box-and-whisker plots of mEPSP amplitude for the same genotypes. The 29°C condition showed a significantly diminished mEPSP distribution profile compared to 25°C (***p < 0.001, Mann-Whitney test).

**Figure 4.**
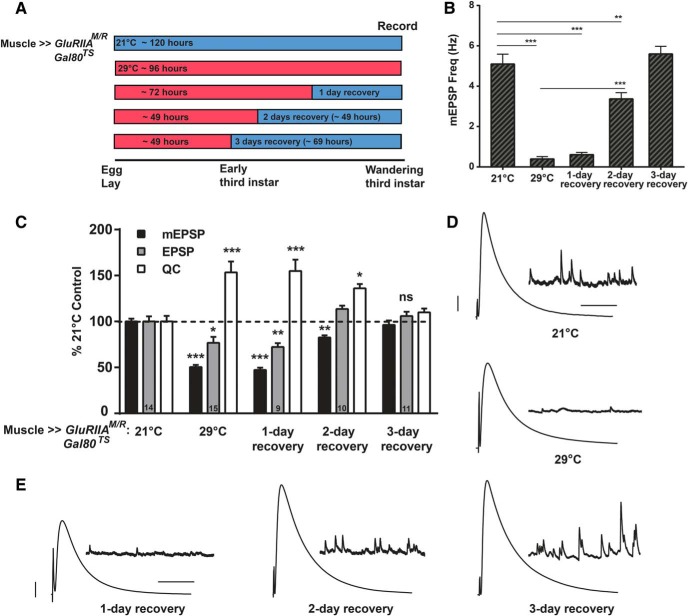
Presynaptic homeostatic potentiation is reversible. ***A***, Diagram of a temperature swap paradigm. Mated animals laid eggs for a small window of 6–8 h. One set of larvae was reared entirely at 21° from egg laying to electrophysiological recording. A second set was raised entirely at 29°C. To test for reversibility of homeostatic potentiation, additional sets were reared initially at 29°C and then swapped to 21°C. Animals were allowed to recover for 1, 2, or 3 d before recording. ***B***, Expression of the dominant-negative transgene throughout life (29°C) caused a dramatic decrease in mEPSP frequency (***p < 0.001, one-way ANOVA with Tukey’s *post hoc*). Frequency remained low after 1 d of recovery at 21°C, compared to baseline 21°C controls, but by 2 d of recovery, the frequency was significantly increased compared to 29°C (***p < 0.001), but still decreased compared to 21°C controls (**p < 0.01). By 3 d of recovery, mEPSP frequency was no different from animals raised entirely at 21°C. ***C***, Normalized electrophysiological data for mEPSP amplitude, EPSP amplitude, and QC for the NMJs of animals raised as described in ***A***. Longer recovery periods yielded electrophysiology that closely approximated the control 21°C rearing condition (all statistics: *p < 0.05, **p < 0.01, ***p < 0.001 compared to 21°C control by one-way ANOVA with Tukey’s *post hoc*). ***D***, Representative electrophysiological traces for 21°C and 29°C. ***E***, Traces for the three recovery conditions. Scale bars for EPSPs (and mEPSPs) are 5 mV (1 mV) and 50 ms (1000 ms) for both ***D*** and ***E***.

**Figure 5.**
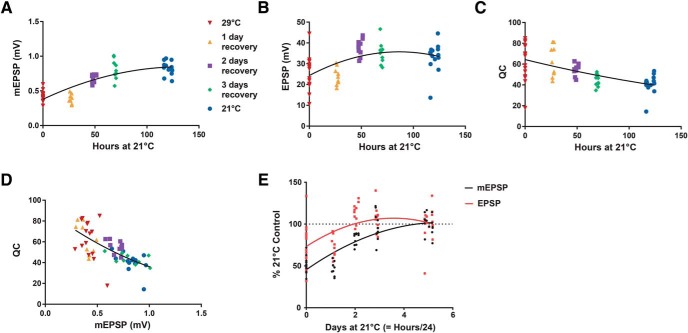
Strong correlations between recovery time and classic homeostatic parameters. Electrophysiological parameter data plotted from the reversibility experiments detailed in Fig. 4. Animals reared for “0 hours” at 21°C are the 29°C condition; animals in the 21°C condition were reared at that temperature for ∼120 h. Each data point is time-locked to the times of the relevant egg-laying period and the recording. ***A***, mEPSP amplitude plotted as a function of hours raised at 21°C. The data show a strong positive correlation between the two parameters (*R* ^2^ = 0.71; nonlinear fit as a quadratic equation). ***B***, EPSP amplitude plotted as a function of hours raised at 21°C. The data show a positive correlation between the two values (*R* ^2^ = 0.28; nonlinear fit as a quadratic equation). ***C***, QC plotted as a function of hours raised at 21°C. The data show a negative correlation between the two values (*R* ^2^ = 0.34; nonlinear fit as a quadratic equation). ***D***, Plots of QC versus mEPSP amplitude for each recording. Consistent with prior reports examining PHP at the NMJ, there was a negative correlation between the two values (*R* ^2^ = 0.47; nonlinear fit as a quadratic equation). ***E***, A comparison of quadratic fits for the mEPSP recovery curve (*y* = –1.835*x* ^2^ + 20.38*x* + 45.41) versus EPSP recovery curve (*y* = –2.648*x* ^2^ + 18.93*x* + 73.18). Both parameters are plotted as a percentage of the 21°C control values, as a function of days (= hours/24) reared at 21°C.

**Figure 6.**
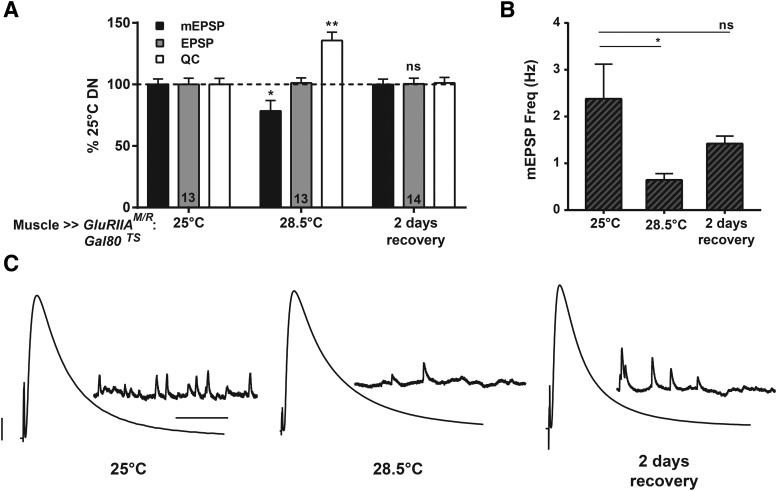
Full homeostatic compensation is reversible. ***A***, Dominant-negative animals with the *Gal80^TS^* transgene raised at 28.5°C showed a significant decrease in mEPSP amplitude (*p < 0.05, compared to 25°C condition; one-way ANOVA with Tukey’s *post hoc*), accompanied by a fully offsetting increase in QC (**p < 0.01). Reversibility of homeostatic potentiation was demonstrated by initially rearing *w; GluRIIA^M/R^/+; BG57-Gal4/Gal80^TS^* animals at 28.5°C for 2 d and swapping them to 25°C for 2 d. mEPSP amplitude, EPSP amplitude, and QC returned to control (25°C throughout life) levels. EPSP amplitudes were virtually identical across all of the conditions. ***B***, Dominant-negative animals raised at 28.5°C throughout life showed a significant decrease in mEPSP frequency (*p < 0.05, one-way ANOVA with Tukey’s *post hoc*). In contrast, the mEPSP frequency in animals after 2 d of recovery was not significantly different from that of the animals reared at 25°C. ***C***, Representative electrophysiological traces. Scale bars for EPSPs (and mEPSPs) are 5 mV (1 mV) and 50 ms (1000 ms).

**Figure 7.**
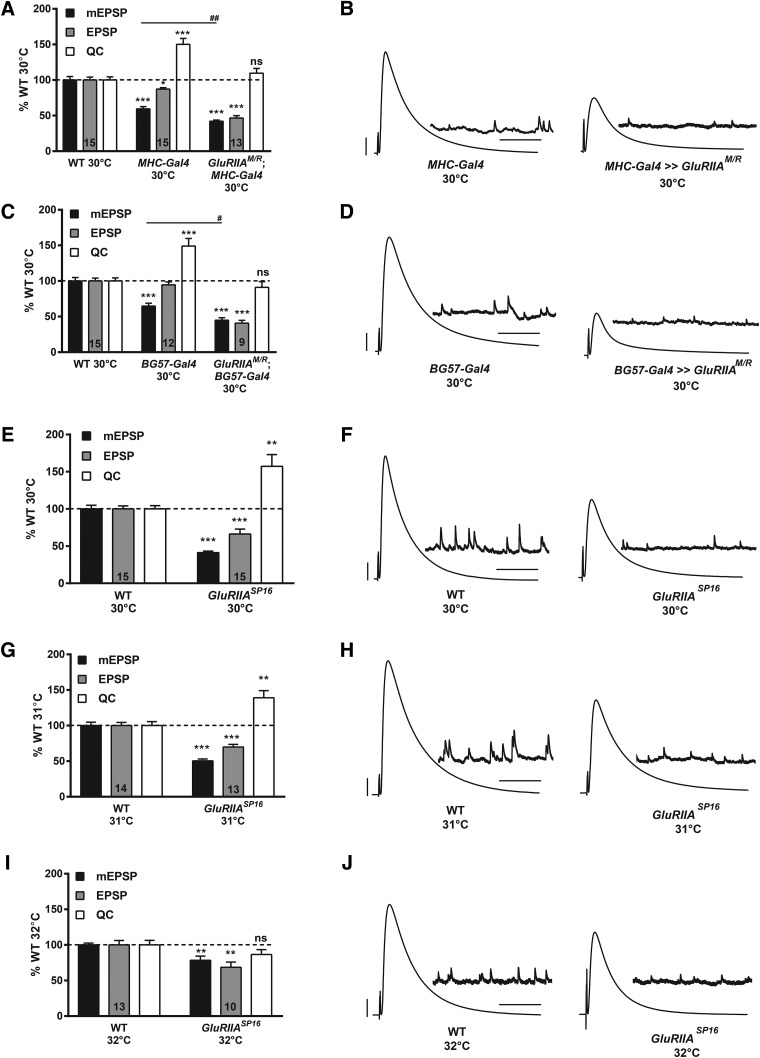
Presynaptic homeostatic potentiation can be impaired at high temperatures. ***A***, WT (*w^1118^*), driver controls (*w;; MHC-Gal4/+*), and dominant-negative NMJs (*w; UAS-GluRIIA^M/R^/+; MHC-Gal4/+*) were compared at 30°C. For driver controls, the mEPSP amplitude was significantly decreased compared to WT (***p < 0.001, one-way ANOVA with Tukey’s *post hoc*), but the EPSP amplitude was only slightly smaller than normal (*p < 0.05 versus WT) because of a homeostatic increase in QC (***p < 0.001 versus WT). For dominant-negative animals, the mEPSP amplitude was markedly decreased (***p < 0.001 versus WT; ##*p* < 0.01 versus driver controls, one-way ANOVA with Tukey’s *post hoc*). A dramatic decrease in EPSP amplitude for the dominant-negative NMJs also occurred (***p < 0.001 versus WT) because there was no significant change in QC (*p* = 0.58 versus WT), indicating a block of PHP. ***B***, Representative electrophysiological traces. Scale bars for EPSPs (and mEPSPs) are 5 mV (1 mV) and 50 ms (1000 ms). ***C***, WT, driver controls (*w;; BG57-Gal4/+*), and dominant-negative animals (*w; UAS-GluRIIA^M/R^/+; BG57-Gal4/+*) were compared at 30°C. For driver controls, the mEPSP amplitude was significantly decreased compared to WT (***p < 0.001, one-way ANOVA with Tukey’s *post hoc*), but the EPSP was normal because of a homeostatic increase in QC (***p < 0.001 versus WT). In contrast, for dominant-negative animals, the mEPSP amplitude was markedly decreased (***p < 0.001 versus WT; #*p* < 0.01 versus driver controls, one-way ANOVA with Tukey’s *post hoc*). A dramatic decrease in EPSP amplitude for the dominant-negative NMJs also occurred (***p < 0.001 versus WT) because there was no significant change in QC (*p* = 0.71), indicating a block of PHP. ***D***, Representative electrophysiological traces. Scale bars as in ***B***. ***E***, *GluRIIA^SP16^* animals and WT controls were raised at 30°C. mEPSPs were significantly decreased for *GluRIIA* mutants (***p < 0.001, Student’s *t* test). EPSP values were also decreased (***p < 0.001) but not to the same magnitude as mEPSP amplitudes because the QC was significantly increased (**p < 0.01). ***F***, Representative electrophysiological traces. Scale bars as in ***B***. ***G***, *GluRIIA^SP16^* animals and WT controls were raised at 31°C. mEPSP and EPSP amplitude were both significantly decreased in *GluRIIA^SP16^* animals (***p < 0.001, Student’s *t* test) and QC was increased (**p < 0.01). ***H***, Representative electrophysiological traces. Scale bars as in ***B***. ***I***, The same genotypes were raised at 32°C. *GluRIIA^SP16^* animals showed a decrease in mEPSP and EPSP amplitude (**p < 0.01, Student’s *t* test), and they failed to show increase in QC (*p* = 0.17). ***J***, Representative electrophysiological traces. Scale bars as in ***B***.

For Figs. 1, 2, and 4–7, wandering third instar larvae were filleted for electrophysiological recordings. For Fig. 3, early third instar larvae were used. In both cases, sharp electrode electrophysiological recordings were taken from muscle 6 of abdominal segments 2 and 3. Briefly, larvae were dissected in a modified HL3 saline comprising NaCl (70 mm), KCl (5 mm), MgCl_2_ (10 mm), NaHCO_3_ (10 mm), sucrose (115 mm = 3.9%), trehalose (4.2 mm = 0.16%), Hepes (5.0 mm = 0.12%), and CaCl_2_ (0.5 mm). Electrophysiological data were collected using an Axopatch 200B amplifier (Molecular Devices) in bridge mode, digitized using a Digidata 1440A data acquisition system (Molecular Devices), and recorded with pCLAMP 10 acquisition software (Molecular Devices). Spontaneous miniature excitatory postsynaptic potential (mEPSP) data were acquired at a sampling rate of 10,000 Hz (one sample/100 μs). The low-pass and high-pass settings were 5 kHz and 1 Hz, respectively. A Master-8 pulse stimulator (A.M.P. Instruments) and an ISO-Flex isolation unit (A.M.P. Instruments) were used to deliver 1-ms suprathreshold stimuli to the appropriate segmental nerve. The average mEPSP amplitude per NMJ was quantified (MiniAnalysis, Synaptosoft), ∼100–200 individual spontaneous release events per NMJ. The noise level for each individual mEPSP event was captured in the same analysis. From this number (noise could be positive or negative when captured by the software), a positive |noise| value was assigned to each mEPSP and averaged across all mEPSPs for a given NMJ. This allowed calculation of both mEPSP noise and mEPSP signal:noise ratios (Table 1). In the case that the mEPSP frequency was extremely low (usually for expression of the dominant-negative glutamate receptor subunit), several minutes of spontaneous recording were done, and all events were measured. Measurements from all NMJs of a given condition were then averaged. For evoked neurotransmission, 30 excitatory postsynaptic potentials (EPSPs) were averaged to find a value for each NMJ. These were then averaged to calculate a value for each condition. Quantal content (QC) was calculated by the ratio of average EPSP and average mEPSP amplitudes for each individual NMJ. An average QC was then calculated for each condition.

**Table 1.** Raw electrophysiological data

Genotype	Condition	mEPSP (mV)	mEPSP noise (µV)	mEPSP/noise ratio	mEPSP frequency (Hz)	EPSP (mV)	QC	I_R_ (MΩ)	RMP (mV)	n	Figure
*w^1118^ (WT)*	25°C	0.85 ± 0.04	16.5 ± 1.8	57.3 ± 5.7	3.5 ± 0.3	32.0 ± 1.2	38.5 ± 1.7	6.4 ± 0.6	–64.6 ± 1.1	13	1, 2
*w;; MHC-Gal4/+*	25°C	0.65 ± 0.03	23.3 ± 1.2	28.2 ± 1.3	4.3 ± 0.5	33.6 ± 1.8	52.1 ± 1.8	4.8 ± 0.2	–66.1 ± 1.2	13	1, 2
*UAS-GluRIIA^M614R^/+; MHC-Gal4/+*	25°C	0.44 ± 0.02	13.8 ± 0.5	32.2 ± 1.7	0.6 ± 0.1	35.0 ± 1.8	80.3 ± 4.1	5.8 ± 0.5	–69.1 ± 1.2	11	1, 2
*w^1118^*	29°C	0.72 ± 0.03	16.7 ± 0.7	43.6 ± 2.2	1.8 ± 0.2	32.9 ± 1.2	46.4 ± 2.2	6.2 ± 0.5	–64.4 ± 0.9	14	1, 2
*w;; MHC-Gal4/+*	29°C	0.52 ± 0.02	21.8 ± 0.7	24.1 ± 1.0	2.4 ± 0.1	31.7 ± 1.3	61.2 ± 2.4	5.3 ± 0.4	–66.5 ± 1.0	12	1, 2
*UAS-GluRIIA^M614R^/+; MHC-Gal4/+*	29°C	0.35 ± 0.02	13.8 ± 0.9	26.4 ± 2.3	0.2 ± 0.0	23.3 ± 1.2	68.9 ± 5.7	5.2 ± 0.2	–65.0 ± 1.1	10	1, 2
*UAS-GluRIIA^M614R^/+; MHC-Gal4/Gal80^TS^*	Early third instar; 25°C	1.27 ± 0.1	26.6 ± 3.1	53.4 ± 7.3	2.0 ± 0.3	33.2 ± 2.1	27.4 ± 2.4	9.5 ± 0.8	–64.7 ± 2.0	10	3
*UAS-GluRIIA^M614R^/+; MHC-Gal4/Gal80^TS^*	Early third instar; 29°C	0.60 ± 0.05	18.7 ± 1.9	32.8 ± 1.2	0.4 ± 0.1	25.1 ± 1.4	43.1 ± 2.6	13.6 ± 0.9	–63.0 ± 0.8	10	3
*UAS-GluRIIA^M614R^/+; MHC-Gal4/Gal80^TS^*	21°C	0.83 ± 0.03			5.1 ± 0.5	33.3 ± 1.9	40.7 ± 2.4	5.6 ± 0.4	–66.3 ± 0.8	14	4, 5
*UAS-GluRIIA^M614R^/+; MHC-Gal4/Gal80^TS^*	29°C	0.42 ± 0.02			0.4 ± 0.1	25.6 ± 2.1	62.5 ± 4.8	6.0 ± 0.3	–66.8 ± 1.1	15	4, 5
*UAS-GluRIIA^M614R^/+; MHC-Gal4/Gal80^TS^*	1-d recovery at 21°C	0.39 ± 0.02			0.6 ± 0.1	24.1 ± 1.4	63.1 ± 5.0	5.3 ± 0.3	–66.0 ± 1.1	9	4, 5
*UAS-GluRIIA^M614R^/+; MHC-Gal4/Gal80^TS^*	2-d recovery at 21°C	0.69 ± 0.02			3.4 ± 0.3	37.8 ± 1.2	55.4 ± 1.9	5.3 ± 0.3	–67.7 ± 0.7	10	4, 5
*UAS-GluRIIA^M614R^/+; MHC-Gal4/Gal80^TS^*	3-d recovery at 21°C	0.80 ± 0.04			5.6 ± 0.4	35.3 ± 1.5	44.7 ± 1.7	5.5 ± 0.2	–68.4 ± 0.9	11	4, 5
*UAS-GluRIIA^M614R^/+; BG57-Gal4/Gal80^TS^*	25°C	0.79 ± 0.04			2.4 ± 0.7	31.1 ± 1.6	39.5 ± 1.9	5.3 ± 0.4	–68.4 ± 1.1	13	6
*UAS-GluRIIA^M614R^/+; BG57-Gal4/Gal80^TS^*	28.5°C	0.62 ± 0.05			0.6 ± 0.1	31.5 ± 1.3	53.6 ± 3.6	7.2 ± 0.4	–67.0 ± 1.3	13	6
*UAS-GluRIIA^M614R^/+; BG57-Gal4/Gal80^TS^*	2-d recovery at 25°C	0.79 ± 0.03			1.4 ± 0.2	31.3 ± 1.4	39.9 ± 1.8	6.3 ± 0.3	–66.7 ± 1.1	14	6
*w^1118^*	30°C	0.78 ± 0.04			2.4 ± 0.2	33.1 ± 1.4	42.9 ± 1.9	6.8 ± 0.3	–66.2 ± 0.8	15	7
*w; GluRIIA^SP16^*	30°C	0.32 ± 0.02			0.8 ± 0.1	21.9 ± 2.2	67.4 ± 6.8	6.0 ± 0.4	–63.0 ± 0.5	15	7
*MHC-Gal4/+*	30°C	0.47 ± 0.02			1.1 ± 0.1	28.9 ± 0.6	64.3 ± 3.6	4.9 ± 0.2	–63.9 ± 0.9	15	7
*UAS-GluRIIA^M614R^/+; MHC-Gal4/+*	30°C	0.33 ± 0.01			0.2 ± 0.0	15.4 ± 1.1	47.0 ± 2.8	5.3 ± 0.2	–64.4 ± 0.9	13	7
*w;; BG57-Gal4/+*	30°C	0.51 ± 0.03			0.6 ± 0.1	31.3 ± 1.4	63.9 ± 4.5	6.6 ± 0.3	–70.2 ± 1.4	12	7
*UAS-GluRIIA^M614R^/+; BG57-Gal4/+*	30°C	0.35 ± 0.03			0.8 ± 0.7	13.5 ± 1.3	39.1 ± 3.4	6.1 ± 0.5	–65.5 ± 1.6	9	7
*w^1118^*	31°C	0.71 ± 0.03			2.4 ± 0.2	36.9 ± 1.6	53.6 ± 3.0	2.0 ± 0.2	–68.1 ± 1.5	14	7
*w; GluRIIA^SP16^*	31°C	0.36 ± 0.02			1.1 ± 0.2	25.8 ± 1.5	74.4 ± 5.4	4.2 ± 0.9	–66.6 ± 1.3	13	7
*w^1118^*	32°C	0.47 ± 0.01			2.4 ± 1.1	30.7 ± 1.9	64.8 ± 4.1	2.0 ± 0.4	–63.7 ± 0.9	13	7
*w; GluRIIA^SP16^*	32°C	0.37 ± 0.03			1.4 ± 0.9	21.0 ± 2.2	56.1 ± 4.4	5.6 ± 0.3	–65.0 ± 0.8	10	7

Full genotypes and rearing conditions for electrophysiological data presented in the study. Average values ± SEM are presented for each parameter; *n*, number of NMJs recorded. Values include mEPSP amplitude, noise, amplitude to noise ratio, and frequency; EPSP amplitude; quantal content (QC); muscle input resistance (I_R_); and resting membrane potential (RMP).

### NMJ immunostaining

Wandering third instar larvae were selected and filleted. Dissected animals were fixed for 4 min in Bouin’s fixative (Ricca Chemical Company), washed using standard procedures, and incubated in primary antibodies at room temperature for 2 h. This was followed by additional washes and another 2-h incubation in secondary antibody at room temperature. Bouton staining was performed to assess NMJ growth by using the following primary antibodies: mouse anti-Synapsin (anti-Syn; 3C11) 1:50 (Developmental Studies Hybridoma Bank) and rabbit anti-Dlg 1:15,000 (Budnik et al. 1996). The following fluorophore-conjugated secondary antibodies were also used (Jackson ImmunoResearch Laboratories): goat anti–mouse 488 1:1000 (DyLight) and goat anti–rabbit 549 1:2000 (DyLight). Larval preparations were mounted in Vectashield (Vector Laboratories) and imaged at room temperature using Zen software on a Zeiss 700 LSM mounted on an Axio Observer.Z1 with an EC Plan-Neofluar 40× Oil DIC Objective (aperture 1.30) or an EC Plan-Apochromat 63× Oil DIC Objective (aperture 1.40; Zeiss). Experimental and control larval preps were imaged using identical acquisition settings, and analyzed using the same procedure and thresholds. Images were prepared for publication in Adobe Photoshop using identical procedures for experimental and control images. Anti-Dlg bouton counts were done by hand in a blinded fashion to quantify synapse growth. For each anti-Dlg–positive bouton counted in muscle, it was verified that there was a corresponding cluster of anti-Syn staining in neurons.

### Statistical analyses

Statistical analyses were conducted using GraphPad Prism software. For normally distributed data, statistical significance was assessed by either Student’s *t* test, when one experimental data set was being directly compared to a control data set, or one-way ANOVA with Tukey’s *post hoc* test when multiple data sets were being compared. Specific *p* value ranges are noted in the figure legends and shown in graphs as *p < 0.05; **p < 0.01; and ***p < 0.001. For some comparisons that potentially trend toward *p* < 0.05 statistical significance but do not achieve it (0.05 < *p* < 0.1), specific values are reported on the graph itself. mEPSP size frequency distributions were analyzed by Kruskal-Wallis test (Fig. 2, comparing three conditions) and Mann-Whitney test (Fig. 3, comparing two conditions) and plotted as cumulative histograms and as box-and-whisker plots. To assess correlations between incubation temperature recovery times and electrophysiological parameters (Fig. 5), nonlinear best-fit curves were generated using quadratic equations. These curves were graphed on top of the raw data, with *R* ^2^ values for the curves reported in the Fig. 5 legend. Most values reported in Table 1 or plotted on bar graphs are mean ± SEM. For box-and-whisker plots, cumulative data are plotted in a box denoting 25th–75th percentiles (bar denotes median; + denotes mean) and whiskers spanning 1st–99th percentiles, and individual extreme data points <1st or >99th percentile are plotted. See Table 2 for summary detail.

**Table 2.** Statistical analysis

Data structure	Type of test or value reported	Confidence intervals
Normal distribution	Student’s *t* test (Figs. 3, 7, 8)	*p< 0.05, **p< 0.01, ***p< 0.001
Normal distribution	One-way ANOVA with Tukey’s *post hoc* (Figs. 1, 2, 4, 6, 7, 8)	*p< 0.05, **p< 0.01, ***p< 0.001
Cumulative distribution	Kruskal-Wallis test (Fig. 2: three data sets)	***p< 0.001
Cumulative distribution	Mann-Whitney test (Fig. 3: two data sets)	***p< 0.001
Nonlinear best-fit curve	*R* ^2^ (Fig. 5)	Not applicable

## Results

### Homeostatic potentiation using a dominant-negative glutamate receptor subunit

A prior study described a transgene encoding a dominant-negative GluRIIA subunit, *UAS-GluRIIA^M614R^*(herein termed *GluRIIA^M/R^* or dominant-negative; DiAntonio et al. 1999). The GluRIIA M614R amino acid substitution resides in the ion conduction pore of the GluRIIA subunit, and it cripples channel function (DiAntonio et al. 1999). Transgenic expression of *UAS-GluRIIA^M/R^* in muscles renders a strong homeostatic challenge (markedly diminished quantal size) and an equally strong compensatory response (increase in quantal content). The prior report demonstrated that evoked amplitudes remain normal as a result of this compensation, presently termed presynaptic homeostatic potentiation (PHP; DiAntonio et al. 1999).

We acquired the dominant-negative transgenic line to test homeostatic reversibility. First, we replicated the published experiments, raising fruit fly larvae at temperatures compatible with the TARGET system that we planned to use to test reversibility (McGuire et al. 2003). We tested 25°C (a common culturing temperature) and 29°C (a temperature at which the GAL80^TS^ protein ceases to inhibit GAL4). We drove *UAS-GluRIIA^M/R^* expression with *MHC-Gal4*, which turns on in first-instar larval muscles (Schuster et al. 1996a, b). We recorded from NMJs of WT, GAL4 driver control, and dominant-negative wandering third instar larvae.

For animals reared at 25°C, WT NMJ electrophysiology was robust and consistent with values canonically reported for WT at this synapse (Fig. 1*A*, *C*, *D*; Table 1 for raw electrophysiological data). For heterozygous *MHC-Gal4/+* driver control NMJs (“*MHC-Gal4*” on figures), we noted a small decrease in quantal size compared to WT (Figs. 1*A*, *C*, *D*). This quantal size decrease was consistent across the entire distribution of the spontaneous miniature excitatory postsynaptic potential (mEPSP) sample (Figs. 2*A*, *B*). Driver controls had a fully offsetting increase in QC, keeping evoked potentials robust (Figs. 1*A*, *C*; Table 1).

Muscle-specific expression of the *UAS-GluRIIA^M/R^* dominant-negative transgene at 25°C caused a stark diminishment of quantal size and frequency compared to controls, evident from a small average mEPSP amplitude and decreased mEPSP amplitudes across the entire distribution (Figs. 1*A*, *C*, and 2*A*, *B*; Table 1). Despite decreased quantal size, there was no diminishment in the average evoked excitatory postsynaptic potential (EPSP) amplitude for dominant-negative NMJs (Figs. 1*A*, *C*, *D*; Table 1)—again, because of a fully offsetting homeostatic increase in QC (Figs. 1*A*, *C*; Table 1). In sum, our recordings at 25°C agreed with the prior finding for the dominant-negative transgene (DiAntonio et al. 1999): full PHP.

For animals raised at 29°C, we garnered similar results as at 25°C, noting a few differences. At 29°C, WT NMJs had slightly smaller mEPSP quantal amplitude and frequency than 25°C WT NMJs (Fig. 1*A–D*; Table 1). This finding was consistent with a prior report measuring NMJ physiology at elevated temperatures (Ueda and Wu, 2015). WT EPSP amplitudes and QC at 29°C were nevertheless robust, similar to the values garnered at 25°C (Fig. 1*A–D*; Table 1). *MHC-Gal4/+* driver controls at 29°C showed slightly diminished average mEPSP amplitudes, a diminishment that persisted across the entire distribution of events (Figs. 1*B*, *C*, and 2*C*, *D*). *MHC-Gal4/+* driver controls at 29°C maintained normal evoked potentials because of homeostatically increased QC (Fig. 1*B–D*).

By contrast, *UAS-GluRIIA^M/R^*-expressing NMJs from 29°C showed a profound decrease in mEPSP size, a partially compensatory increase in QC compared to WT, and markedly reduced quantal frequency compared to WT (Figs. 1*B*, *C*, and 2*C*, *D*). Although QC was significantly increased in dominant-negative NMJs compared to WT, it did not increase enough to bring average dominant-negative EPSP amplitudes at 29°C fully back to WT or *MHC-Gal4/+* control levels (Fig. 1*B–D*).

The diminished mEPSP frequency for dominant-negative-expressing NMJs (Fig. 1*C*) is an interesting phenotype; it mirrors numerous cases at the *Drosophila* NMJ in which there is greatly diminished glutamate receptor subunit expression or function (Featherstone et al. 2005; Daniels et al. 2006; Kim et al. 2012, 2015; Brusich et al. 2015; Ramos et al. 2015). Given the very low observed mEPSP frequencies for dominant-negative NMJs, one potential concern was a severe underestimation of QC in these recordings due to unobserved mEPSP events that could have been lost in the noise of the recordings. In theory, such lost events could confound analyses of homeostatic compensation. We calculated the average noise for mEPSP recordings from every genotype, as well as an mEPSP size/mEPSP noise ratio (Table 1). Noise varied slightly from genotype to genotype, but it was vanishingly small for each one (∼0.02 mV/mEPSP for each; Table 1). This small noise yielded very large mEPSP/noise ratios for all genotypes (Table 1). Those ratios did vary because of different average quantal sizes between the genotypes; as a result, the ratio was a little smaller for dominant-negative NMJs than for WT controls (Table 1). Importantly, however, dominant-negative GluRIIA^M/R^-expressing NMJs showed similar values by these noise measures at 25°C versus 29°C (Table 1). This indicated that recording noise was not a factor in determining full versus partial homeostatic compensation between the 25°C and 29°C conditions.

### Early third instar larvae express homeostatic plasticity

Our initial experiments showed that it is possible to observe increases in QC for *UAS-GluRIIA^M/R^*-expressing animals compared to WT, when raising flies at either 25°C or 29°C. We sought to test homeostatic reversibility. The TARGET system augments GAL4/UAS expression, adding a ubiquitously expressed *Tubulin_P_*-*Gal80^TS^* GAL4 inhibitor transgene (McGuire et al. 2003). We added this GAL80^TS^ expression feature to the *MHC-Gal4* ≫ *UAS-GluRIIA^M/R^* genetic background. The temperature sensitivity of the GAL80^TS^ protein permits tight control over when GAL4-responsive transgenes are expressed (McGuire et al. 2003). We predicted that the dominant-negative transgene should be repressed by GAL80^TS^ at low temperatures; conversely, *UAS-GluRIIA^M/R^* should be actively expressed when GAL80^TS^ is inactive (∼29°C or higher; McGuire et al. 2003). To study the reversibility of PHP, animals could be reared at high temperature and then swapped to a lower temperature at an appropriate developmental time point.

An ideal temperature swap point would be late enough to detect PHP but early enough to allow recovery time after cessation of the dominant-negative *UAS-GluRIIA^M/R^* expression. We crossed *UAS-GluRIIA^M/R^*; *Tub_P_-Gal80^TS^* × *MHC-Gal4* stocks. Mated animals were transferred to 25°C or 29°C for egg laying and subsequent larval development. We selected early third instar progeny for electrophysiological recording. At temperature ranges of 25°C–29°C, early third instar larvae develop roughly 48–60 h after egg-laying. We staged these small animals unambiguously by examining their posterior spiracles for an orange-colored tip.

For animals raised entirely at 25°C, early third instar NMJ mEPSP size was large—significantly larger than one would observe for third instar larvae (Fig. 3*A*, *B*; Table 1; compare with Fig. 1). This was expected because small muscles have a significantly greater input resistance and enhanced quantal size (Lnenicka and Mellon, 1983a, b; Davis and Bezprozvanny, 2001; Table 1). Nevertheless, early third instar larval NMJs raised at 25°C showed normal evoked amplitudes, consistent with a stable level of evoked muscle excitation throughout development (Fig. 3*A*, *B*; Table 1; compare with Fig. 1).

We expected that genotypically equivalent early third instars raised entirely at 29°C would express the dominant-negative transgene. NMJs from these 29°C animals showed sharply reduced average mEPSP amplitude and frequency compared to their stage- and size-matched counterparts raised at 25°C (Fig. 3*A*, *B*; Table 1), and this mEPSP amplitude decrease persisted throughout the distribution of events (Fig. 3*C*, *D*). There was a robust increase in quantal content at 29°C, resulting in EPSP amplitudes that were nearly normal, but not quite at the same level as at 25°C. Therefore, as with the earlier experiments, PHP for dominant-negative early third instar NMJs raised exclusively at 29°C was present, but not full (Fig. 3*A*, *B*; Table 1). Again, as a quality-check control, absolute mEPSP noise levels were small for both conditions (Table 1), and the mEPSP size/mEPSP noise ratios were similar to values obtained for the prior experiment (Table 1).

### Partial homeostatic potentiation is reversible

At 29°C, PHP was not full, but QC increases versus controls were robust, still making it possible to test homeostatic reversibility. We generated additional *MHC-Gal4* ≫ *UAS-GluRIIA^M/R^* larvae with the *Tub_P_-GAL80^TS^*transgene. For reversibility experiments, we chose 21°C as a permissive GAL80^TS^ shift temperature because 21°C permitted multiple electrophysiological time point measurements over a long recovery window (Fig. 4*A*).

Control (no PHP) animals raised entirely at 21°C took ∼120 h after egg laying to reach the wandering third instar stage, whereas control (PHP) animals raised entirely at 29°C took ∼96 h to reach the same stage (Fig. 4*A*). A 1-d recovery condition was used, exposing animals to the *UAS-GluRIIA^M/R^* challenge for ∼72 h (29°C) and allowing them to recover at 21°C for 1 d before recording. We also tested 2- and 3-d recovery conditions, rearing larvae at 29°C for ∼49–50 h and then swapping them to 21°C until they reached wandering third instar stage. Because of the length of the egg lays and small variations in developmental time, some animals reached the wandering third instar recording stage after ∼2 d at 21°C, and others took closer to 3 d (Fig. 4*A*).

Control *MHC-Gal4* ≫ *UAS-GluRIIA^M/R^* larvae with ubiquitously expressed *Tub_p_-Gal80^TS^* and raised at 21°C showed neurotransmission indistinguishable from WT control animals reared at 25°C (compare Figs. 1 and 4; Table 1). By contrast, genetically identical control animals raised at 29°C throughout life showed electrophysiological phenotypes similar to dominant-negative *MHC-Gal4* ≫ *UAS-GluRIIA^M/R^* animals raised at 29°C (compare Figs. 1 and 4; Table 1). Compared to counterparts raised at 21°C, animals raised at 29°C showed reduced mEPSP frequency, reduced mEPSP size, slightly below-normal EPSP amplitudes, and increased QC, indicating robust PHP (Fig. 4*B*, *C*; Table 1).

Recovery conditions showed physiologic signatures that corresponded with how much time was spent at 21°C. Compared to the 29°C condition, the 1-d 21°C recovery condition showed no significant changes in physiologic properties and no reversal of PHP (Fig. 4*B–E*). By contrast, the 2-d recovery condition showed intermediate physiology. Compared to constant exposure to 21°C, the 2-d recovery condition still had diminished mEPSP amplitude and frequency, but not nearly as diminished as constant exposure to 29°C (Fig. 4*B–E*). Interestingly, the 2-d recovery EPSP amplitudes revealed fully restored levels of excitation, due to an offsetting QC level (Fig. 4*C–E*). The 3-d recovery condition showed electrophysiology that was not significantly different from the constant 21°C condition (Fig. 4*B–E*), indicating a full reversal of PHP.

We further analyzed the aggregate data from the reversibility experiment. We wished to analyze reversal parameters related to recovery time. We plotted mEPSP (Fig. 5*A*), EPSP (Fig. 5*B*) and QC (Fig. 5*C*) values versus the number of hours animals spent at the recovery temperature, with a specific recovery time value for each NMJ, time-locked to the egg laying period and the recording time after recovery/development at 21°C. Each plot showed hallmarks of PHP reversal: mEPSP and EPSP amplitudes positively correlated with time at 21°C (Fig. 5*A*, *B*), and QC values inversely correlated with time at 21°C (Fig. 5*C*). Consistent with the observation that PHP was present (but not full) at 29°C, individual data points from NMJs of animals raised entirely at 29°C showed a wide variability of QC values (Fig. 5*C*). Prior studies of homeostatic plasticity at the *Drosophila* NMJ have shown that by analyzing hundreds of individual recording values, QC inversely scales with quantal size across genotypes, and as a result, evoked excitation levels remain stable (Frank et al. 2006; Davis and Müller, 2015; Gaviño et al. 2015). For our temperature swap experiments—this time conducting a comparison within a single genotype—this also proved to be the case (Fig. 5*D*). Finally, we checked whether there was a leading component to the recovery, testing whether mEPSP and EPSP values recovered at similar or different rates. We coplotted each parameter’s recovery curve relative to the 21°C control values and fitted the nonlinear curves with quadratic functions. Both parameters appear to recover at similar rates, with the EPSP values reaching the 21°C baseline level first (Fig. 5*E*).

### Full homeostatic potentiation is also reversible

Homeostatic potentiation was robust, yet partial, at the 29°C condition. We hoped to test a condition that was both compatible with full PHP and the reversibility assay. We attempted a new temperature swap, changing parameters. We reasoned that by making the environmental challenge to the NMJ less harsh—by using a lower temperature, putting forth a less severe level glutamate receptor loss, or both—we would be able to generate an ideal full PHP reversibility test condition. For one change, we lowered the restrictive GAL80^TS^-impairing temperature from 29°C to 28.5°C. Other studies using the TARGET system in *Drosophila* have reported that 28.5°C is somewhat effective at impairing GAL80^TS^ function (Staley and Irvine, 2010; Corrigan et al. 2014; Redhai et al. 2016). Second, we replaced the *MHC-Gal4* muscle driver with the *BG57-Gal4* muscle driver (Budnik et al. 1996). Finally, we tried 25°C as the permissive condition.

We generated new sets of larvae for this swap experiment: *BG57-Gal4* ≫ *UAS-GluRIIA^M/R^* larvae with the *TubP-Gal80^TS^* transgene. As expected, animals raised at 25°C throughout life (GAL80^TS^ on) developed NMJs with electrophysiological properties similar to other control conditions already reported (Fig. 6*A*, *C*; Table 1). Also as expected, animals raised at 28.5°C throughout life (GAL80^TS^ impaired) had significantly diminished NMJ mEPSP amplitudes (Fig. 6*A*; Table 1). Of note, the 28.5°C NMJs had markedly diminished mEPSP frequency (Fig. 6*B*), which is an indication of successful expression of the dominant-negative *GluRIIA^M/R^* transgene (Fig. 1). NMJs from those 28.5°C animals also showed completely normal EPSP amplitudes because of a full, offsetting homeostatic increase in QC (Fig. 6*A*, *C*; Table 1). Finally, animals raised at 28.5°C until early third instar and then swapped to 25°C for the final 2 d of larval development showed NMJ electrophysiology indistinguishable from that of animals raised at 25°C throughout life, indicating a complete reversal of PHP (Fig. 6).

### Homeostatic potentiation induced by glutamate receptor function loss is impaired at high temperatures

*UAS-GluRIIA^M/R^* transgene-induced homeostatic potentiation was robust, yet partial, at the 29°C condition. We wondered whether PHP might specifically be impaired at the NMJ when flies are raised at high temperatures. An alternative idea is that a functional limit of the homeostatic signaling system could be due to a dual combination of factors challenging synapse function, such as high temperature combined with glutamate receptor function loss.

To test this idea further, we drove the dominant-negative transgene in the muscle, setting up crosses to generate both *MHC-Gal4* ≫ *UAS-GluRIIA^M/R^* and *BG57-Gal4* ≫ *UAS-GluRIIA^M/R^* animals, as well as driver-specific controls. For the driver controls, quantal size was somewhat diminished at 30°C (Fig. 7*A–D*; Table 1). This was consistent with the idea that quantal size is generally diminished at very high temperatures (Ueda and Wu, 2015), although taken together with our prior results (Fig. 1), it also seemed likely that simply driving GAL4 protein in the muscle can cause this phenotype. Despite diminished quantal size, evoked EPSP amplitudes were still robust for 30°C driver controls because of homeostatic increases in QC (Fig. 7*A–D*; Table 1). For the dominant-negative NMJs, there was a significant reduction in mEPSP size beyond what was measured for the driver controls. Moreover, evoked amplitudes were weak, diminished significantly versus driver controls because of no significant increase in QC versus WT controls (Fig. 7*A–D*; Table 1). These data indicated that signaling processes that maintain PHP in response to the dominant-negative transgene may break down at high temperatures.

We were uncertain whether this high-temperature failure in PHP was due to the expression levels of the dominant-negative glutamate receptor subunit at 30°C, the high temperature, or a combination of both. High temperature alone seemed unlikely, because the 30°C driver controls also had diminished quantal size accompanied by robust PHP. Glutamate receptor function loss alone also seemed unlikely, because quantal size for the dominant-negative NMJs was not starkly diminished at 30°C compared to earlier experiments run at 25°C and 29°C (Fig. 1; Table 1).

To extend our inquiry, we examined an additional homeostatic challenge to NMJ function. We raised WT and *GluRIIA^SP16^* null deletion flies (Petersen et al. 1997) at 30°C, 31°C, and 32°C. WT NMJs showed relatively normal physiology at 30° and 31°C (Fig. 7*E–H*; Table 1). Interestingly, NMJs from *GluRIIA^SP16^* deletion animals raised at 30° and 31°C showed significant PHP, with reduced mEPSP amplitudes and robust increases in QC compared to WT (Fig. 7*A*, *B*; Table 1). However, we noted that *GluRIIA^SP16^* EPSP amplitudes were reduced compared to WT, meaning that the PHP was only partial (Table 1). These *GluRIIA^SP16^* QC data (Fig. 7*E*, *G*) and the 30°C muscle-driver control QC data (Fig. 7*A*, *C*) argue against the notion that high temperature alone completely impairs PHP. Temperature could still be a contributing factor.

We next raised WT and *GluRIIA^SP16^* null animals at 32°C. This extreme temperature was suboptimal for animal health. WT animals, while able to reach the third instar stage, had poor muscle health, as measured by low input resistance and low mEPSP amplitude compared to conditions of normal rearing temperatures (Table 1). Nevertheless, 32°C WT NMJs had evoked events that were nearly normal in size (Fig. 7*I*, *J*; Table 1). Homozygous *GluRIIA^SP16^* larvae that survived to the wandering third instar stage at 32°C were rare. For the 32°C *GluRIIA^SP16^* homozygotes that did survive, the NMJs showed a further decrease in mEPSP amplitude and no PHP expression (Fig. 7*I*, *J*; Table 1). Taken together, the electrophysiological data are consistent with the idea that a dual challenge—glutamate receptor function loss plus extreme high temperature—abolishes PHP.

### Dual-challenged NMJs show diminished growth at high temperatures

**Figure 8.**
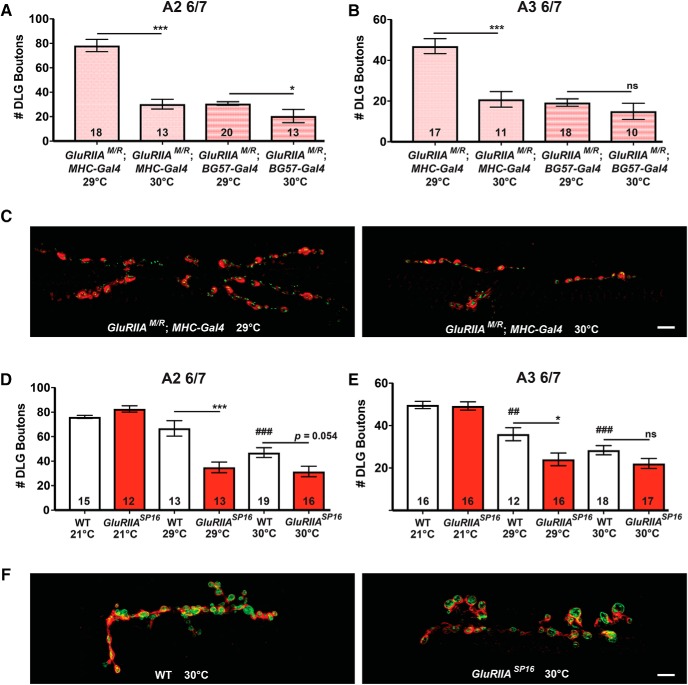
NMJ undergrowth correlates with glutamate receptor function loss plus high temperature. ***A***, *w; UAS-GluRIIA^M/R^/+; MHC-Gal4/+* and *w; UAS-GluRIIA^M/R^/+; BG57-Gal4/+* animals were raised at 29°C or 30°C and costained with anti-DLG (red) and anti-Synapsin antibodies (green) to visualize synaptic boutons. Boutons were counted based on postsynaptic DLG staining and double checked for presynaptic Synapsin. At abdominal segment A2 muscle 6/7, *w; UAS-GluRIIA^M/R^/+; MHC-Gal4/+* animals raised at 30°C showed a significant decrease in bouton number compared to genetically identical counterparts raised at 29°C (***p < 0.001, Student’s *t* test). *w; UAS-GluRIIA^M/R^/+; BG57-Gal4/+* animals raised at 30°C showed only a slight decrease in bouton number compared to counterparts at 29°C (*p < 0.05), although in this case, the 29°C NMJs already showed stunted growth. ***B***, Boutons were also quantified at synapse A3 6/7. *w; UAS-GluRIIA^M/R^/+; MHC-Gal4/+* animals raised at 30°C showed a significant decrease in bouton number compared to counterparts raised at 29°C (***p < 0.001). There was no significant difference between 29°C and 30°C for *w; UAS-GluRIIA^M/R^/+; BG57-Gal4/+* animals; again, both conditions for this genotype showed severely stunted NMJ elaboration. ***C***, Representative NMJ images of synapse A2 6/7 for *w; GluRIIA^M/R^/+; MHC-Gal4/+* animals raised at 29°C and 30°C. Scale bar, 10 μm. ***D***, WT and *GluRIIA^SP16^* animals were raised at 21°C, 29°C, or 30°C and stained with anti-DLG (red) and anti-Synapsin (green). At 29°C, *GluRIIA^SP16^* animals showed a significant decrease in bouton number compared to 29°C WT controls (***p < 0.001, one-way ANOVA with Tukey’s *post hoc*). Additionally, WT NMJs raised at 30°C showed decreased growth compared to WT counterparts raised at 21°C (###*p* < 0.001). ***E***, DLG boutons were quantified at abdominal segment 3 for the same genotypes. *GluRIIA^SP16^* NMJs at 29°C showed a decrease in bouton number compared to WT at the same temperature (*p < 0.05). Additionally, WT NMJs raised at 29°C (##*p* < 0.01) and 30°C (###*p* < 0.001) showed decreased growth compared to the 21°C condition. ***F***, Representative images of synapse A2 6/7 WT and *GluRIIA^SP16^* NMJs at 30°C, showing few boutons and a disorganized anti-Synapsin pattern. Scale bar, 10 μm.

We asked whether synaptic growth defects might correlate with PHP deficits when raising glutamate receptor–compromised animals at high temperatures. We took larvae raised in a subset of the high temperature conditions described (Fig. 7) and immunostained NMJs with anti-Synapsin (Syn, presynaptic vesicles) and anti-Discs Large (Dlg, postsynaptic density) antibodies to assess NMJ development by immunofluorescence. We quantified synaptic bouton elaboration. For dominant-negative glutamate receptor expression under *MHC-Gal4* control, there was a sharp decrease in NMJ growth at 30°C compared to the 29°C condition (Fig. 8*A–C*). By contrast, *BG57-Gal4*–driven dominant-negative expression elicited sharply diminished growth at both 29°C and 30°C (Fig. 8*A*, *B*).

For WT and *GluRIIA^SP16^*, we collected NMJ growth data over a range of temperatures: 21°, 29°, and 30°C. At 21°C, growth was similar between the two genotypes (Fig. 8*D*, *E*). At 29°C, an impairment of NMJ growth in *GluRIIA^SP16^* nulls was apparent, whereas WT NMJ growth was still relatively normal (Fig. 8*D*, *E*). At 30°C, both WT and *GluRIIA^SP16^* nulls showed diminished overall growth (Fig. 8*D–F*), with *GluRIIA^SP16^* growth numerically (but not statistically) diminished. At 30°C for these genotypes, the anti-Syn staining had a tendency to appear disorganized, spreading out over a larger area in the presynaptic bouton (Fig. 8*F*). Collectively, these data are consistent with the idea that at high temperatures under conditions of glutamate receptor loss, PHP failure correlates with NMJ growth and development defects. It is uncertain whether these growth parameters are causally related to the limit in homeostatic signaling.

## Discussion

For the *Drosophila* NMJ, homeostatic potentiation is a robust and sensitive process. One assumption supported by all available data is that the larval NMJ is capable of modulating its vesicle release at any time point during development, in accordance with the presence or absence of a homeostatic challenge to synapse function. Rapid, acute induction of homeostatic signaling has been demonstrated at the *Drosophila* NMJ by application of PhTox to impair the glutamate receptors (Frank et al. 2006), but the reversibility of this particular modulation (or any other modulation at the *Drosophila* NMJ) had not been studied. More generally, it is not clear what happens in metazoan nervous systems when strong perturbations that induce homeostatic signaling are introduced for long periods of developmental time and then later removed.

We present evidence that PHP at the *Drosophila* neuromuscular synapse is a reversible process. In doing so, we confirm prior findings showing that there is a tight inverse relationship between quantal amplitude and QC at the NMJ (Fig. 5). We complement those findings with the results of temperature shift experiments. We find that PHP is measurable at an early stage of larval development (Fig. 3) and can be erased over a matter of days (Figs. 4–6). Interestingly, at high temperatures, PHP induced by impairing glutamate receptor function either fails or falls short of full compensation (Figs. 1, 3, 4, 7). This failure appears to correlate with impaired NMJ growth in the same animals (Fig. 8).

### Why is reversibility slow after dominant-negative GluRIIA^M/R^ removal?

There was a robust expression of PHP for NMJs of *MHC-Gal4* ≫ *UAS-GluRIIA^M/R^* larvae with the *Tub_P_-Gal80^TS^* transgene raised at 29°C for 48 h after egg-laying (Fig. 3). Once the expression of the dominant-negative *UAS-GluRIIA^M/R^* transgene was halted, this expression of PHP was erased over a slow 48- to 72-h period (Fig. 4).

If PHP is a readily reversible homeostatic process, why is there a days-long time lag to reverse it? Our data likely reflect a constraint of the dominant-negative GluRIIA^M/R^ experimental perturbation, rather than the NMJ’s capacity to respond quickly to the changed environment. In a prior study, researchers expressed functional, tagged GluRIIA transgenic subunits at the NMJ and performed fluorescence recovery after photobleaching (FRAP) experiments (Rasse et al. 2005). Those experiments demonstrated that receptor turnover rates at the *Drosophila* NMJ are extremely slow: it appears that once postsynaptic densities (PSDs) reach a critical size, GluRIIA subunits are stably incorporated (Rasse et al. 2005). For our study, this likely means that the temperature downshifts in the reversibility experiments (Figs. 4 and 6) represented an opportunity for the NMJ to incorporate endogenous WT GluRIIA into a significant number of new PSDs while it continued to grow (Rasse et al. 2005; Schmid et al. 2006, 2008). Given sufficient growth, the endogenously expressed GluRIIA would gradually overcome the previously incorporated dominant-negative GluRIIA^M/R^ subunits. This would restore electrophysiological parameters to normal levels, which is consistent with our data.

### Reversibility of rapid and sustained forms of homeostatic plasticity

The majority of recent studies about synaptic homeostasis at the *Drosophila* NMJ have emphasized that presynaptic adjustments to neurotransmitter release properties must occur within minutes of drug-induced (PhTox) postsynaptic receptor inhibition to induce a rapid and offsetting response to PhTox challenge. Some important parameters uncovered through these studies include regulation of presynaptic Ca^2+^ influx (Frank et al. 2006, 2009; Müller and Davis, 2012; Younger et al. 2013; Wang et al. 2014, 2016a); regulation of the size of the readily releasable pool (RRP) of presynaptic vesicles (Weyhersmüller et al. 2011; Harris et al. 2015; Müller et al. 2015, 2012; Wang et al. 2016a); control of presynaptic SNARE-mediated fusion events (Dickman and Davis, 2009; Müller et al. 2011; Dickman et al. 2012); control of neuronal excitability (Bergquist et al. 2010; Younger et al. 2013; Parrish et al. 2014; Orr et al. 2017b); and recently, implication of endoplasmic reticulum calcium-sensing activities (Genç et al. 2017; Kikuma et al. 2017); presynaptic glutamate receptor activity (Kiragasi et al. 2017); and finally, identification of a retrograde, trans-synaptic signaling system governed by the Semaphorin 2b ligand and the Plexin B receptor (Orr et al. 2017a).

For almost all of the cases in which a mutation or an experimental condition blocks the short-term induction of homeostatic signaling, the same perturbation has also been shown to block its long-term maintenance. Interestingly, however, the converse is not true. Additional studies have shown that the long-term consolidation (or expression) of homeostatic signaling at the NMJ can be genetically uncoupled from its induction. Select molecules seem to be dedicated to a long-term maintenance program that involves protein translation and signaling processes in both the neuron and the muscle (Frank et al. 2009; Marie et al. 2010; Penney et al. 2012; Brusich et al. 2015; Kauwe et al. 2016; Spring et al. 2016). Recent data suggest that such long-term processes may take 6 h or more to take full effect (Kauwe et al. 2016).

As more molecular details about HSP are elucidated, it will be interesting to test whether the rapid induction and sustained consolidation of PHP can be reversed by similar or separate mechanisms, and what the time courses of those reversal mechanisms are. At the mouse NMJ, reversibility was recently demonstrated pharmacologically. d-Tubocurarine was applied at a subblocking concentration to impair postsynaptic acetylcholine receptors. Within seconds of drug application, QC increased—and then within seconds of drug washout, it decreased again (Wang et al. 2016b). Follow-up experiments suggested that those rapid, dynamic changes in PHP dynamics at the mouse NMJ were mediated by a calcium-dependent increase in the size of the RRP of presynaptic vesicles (Wang et al. 2016b). Because there seem to be several similarities between the mouse NMJ and the *Drosophila* NMJ (Frank, 2014; Davis and Müller, 2015), it is possible that PHP at the insect NMJ can also be rapidly reversed.

It is instructive to consider mammalian synaptic preparations and study how homeostatic forms of synaptic plasticity are turned on and off. Groundbreaking work on cultured excitatory vertebrate synapses showed that in response to activity deprivation (or promotion), synapses employ scaling mechanisms by adding (or subtracting) AMPA-type glutamate receptors to counteract the perturbation (O’Brien et al. 1998; Turrigiano et al. 1998). Bidirectional scaling suggested that reversible mechanisms likely dictate homeostatic scaling processes. Complementary studies testing scaling reversibility have borne out this prediction (Rutherford et al. 1997; Swanwick et al. 2006; Wang et al. 2011). Additionally, evidence for reversible forms of homeostatic scaling have been found in rodent sensory systems, such as auditory synapses after hearing deprivation (and restoration to reverse; Whiting et al. 2009) and in the visual cortex after light deprivation (and restoration to reverse; Goel and Lee, 2007). Collectively, the vertebrate and invertebrate studies support the notion that reversible fine-tuning is an efficient strategy used to stabilize activities in metazoan nervous systems. One advantage offered by the *Drosophila* system is a genetic toolkit to uncover possible reversibility factors.

### Partial or failed homeostatic signaling at high temperatures

Are there environmental limitations for homeostatic potentiation at the *Drosophila* NMJ? Clearly there are. Our data suggest that a combination of high temperature (30°C–32°C) plus impaired glutamate receptor function can severely limit the NMJ’s ability to compensate for reduced neurotransmitter sensitivity. High temperature alone does not seem to be a severe enough restriction to impair PHP, because *Gal4* driver controls at 30°C have somewhat reduced quantal size, but a fully (or nearly fully) offsetting increase in QC (Fig. 7*A–*D). Likewise, reduced glutamate receptor function alone does not appear to be a sufficient barrier to impair PHP. For example, quantal size is severely diminished when the dominant negative *UAS-GluRIIA^M/R^* transgene is expressed at 25°C, but PHP is nevertheless intact (Fig. 1*A*).

It is not clear what the molecular or anatomic basis of this limit on PHP is. We do know that it is not an issue of NMJ excitation at high temperatures, because evoked neurotransmission for WT (or driver control) NMJs remains remarkably robust over a range of temperatures, including 30°C (Table 1). Nor does it seem to be an elimination of PHP in general, because PHP was still present in the case of *GluRIIA^SP16^* animals raised at 30°C (Fig. 6; Table 1), revealing a distinction between the null *GluRIIA* condition and the dominant-negative *GluRIIA* condition. The limitation seems to be on homeostatic signaling that supports PHP at high temperatures in the face of the dominant-negative transgene expression.

Temperature effects on neurophysiology are well documented. Recent work in crustaceans demonstrates that robust and reliable circuits such as the neurons driving the rhythmicity stomatogastric nervous system can fail under extreme temperature challenges (Tang et al. 2012; Rinberg et al. 2013; Marder et al. 2015). For the *Drosophila* NMJ, prior studies of larval development documented a significant enhancement of synaptic arborization when larvae were raised at higher temperatures (Sigrist et al. 2003; Zhong and Wu, 2004). Additional studies have shown that NMJ growth plasticity can be additionally affected by mutations that affect neuronal excitability (Budnik et al. 1990; Zhong et al. 1992; Lee and Wu, 2010). Given the backdrop of these data, it is not unreasonable to hypothesize that the tolerable limits of synaptic activity challenge could be different at different temperatures.

For our experiments, 29°C–32°C represents a potential failure zone for homeostatic potentiation at the *Drosophila* NMJ. We must note that our data suggest that the coping capacity of the NMJ is dependent on genotype. WT NMJs cope at all temperatures. By contrast, for dominant-negative GluRIIA-expressing NMJs, 29°C is a point at which PHP becomes partial (Figs. 1 and 2), and 30°C is a point at which it fails (Fig. 6). For *GluRIIA^SP16^* subunit deletion NMJs, there is robust, but partial PHP at 30°C (Fig. 7), not unlike the compensation seen for the dominant-negatives at 29°C. *GluRIIA^SP16^* NMJs fail to execute PHP only when temperature is pushed to an extreme range, such as 32°C. Why do these differences persist? It is not clear. The answer could relate to the well-documented temperature-induced alterations in NMJ growth, or alternatively, a limited availability of synaptic factors that are needed to cope with a double challenge of high temperature and particular impairment of glutamate receptor function. Future molecular and physiologic work will be needed to unravel those possibilities in the contexts of different genetic backgrounds and culturing conditions.

## Synthesis

Reviewing Editor: Pablo Castillo, Albert Einstein College of Medicine Decisions are customarily a result of the Reviewing Editor and the peer reviewers coming together and discussing their recommendations until a consensus is reached. When revisions are invited, a fact-based synthesis statement explaining their decision and outlining what is needed to prepare a revision will be listed below. The following reviewer(s) agreed to reveal their identity: Martin Müller, Lu Chen

Your manuscript has been reviewed by two experts in the field. Both of them agree on the good quality of the results and the significance of your study in demonstrating reversibility of homeostatic plasticity in vivo. However, as you will see below, they raise significant criticisms regarding the interpretation of the results, and point out important internal inconsistencies that need to be fully addressed in your revised manuscript.

REVIEWER #1

In the present study, the authors test if homeostatic potentiation of release in response genetic glutamate receptor perturbation at the Drosophila NMJ is a reversible and temperature-sensitive process. Taking advantage of the inducible Gal4/Gal80ts/UAS expression system, they use muscle-specific expression of a previously published dominant-negative glutamate receptor subunit (GluRIIAm614r) to investigate reversibility. First, they confirm that muscle-specific expression of GluRIIAm/r induces an increase in release upon temperature-dependent inactivation of Gal80ts. The increase in release was more pronounced if the 3rd instar mutant larvae were raised at a restrictive temperature of 25{degree sign}C as compared to 29{degree sign}C. After demonstrating an increase in release upon GluRIIAm/r expression at the early third instar larval stage, GluRIIAm/r was expressed at the restrictive temperature of 29{degree sign}C for up to 96 hours with recovery periods at the permissive temperature (21{degree sign}C) between 24 and 69 hours. The major finding of the study is that mEPSP amplitude and quantal content fully return to baseline control levels within 48-72 hours after repressing GluRIIAm/r expression. In a final set of experiments, the authors uncover that GluRIIAm/r expression at 30{degree sign}C disrupts homeostatic potentiation of release. By contrast, GluRIIAsp16 mutant synapses express homeostatic potentiation at the same temperature.

Despite numerous studies that have taken advantage of the Drosophila NMJ to link genes to homeostatic plasticity, it had remained elusive if homeostatic plasticity at this synapse meets an important requirement of truly homeostatic control systems: reversibility. Thus, the study addresses an important question. The experiments are well designed, the data are solid and support most of the conclusions. I therefore consider this work generally suitable for publication in eNeuro. However, the authors need to address the following points to strengthen the data and some of the major conclusions.

MAJOR POINTS

1. The authors did not investigate the reversibility/temperature sensitivity of the acute induction of homeostatic plasticity induced by pharmacological glutamate receptor perturbation. This should be reflected in the title (e.g. "Long-term expression of homeostatic plasticity")

2. Why is the genetic control in figure 1 w1118 and not Gal-4/+ and/or UAS/+? Could the authors please comment on this/report the respective values?

3. mEPSP signal-to-noise ratio: As noted by the authors the decrease in mini frequency in GluRIIA mutants (Fig. 1C) is likely due to the fact that a significant fraction of mEPSPs is lost in the noise (can the authors exclude presynaptic effects?). This, in turn, suggests that the authors overestimate mini amplitude/underestimate the decrease upon GluR inhibition. This effect is expected to be more pronounced with smaller minis amplitudes. Based on this - could the "non-perfect" homeostatic increase in quantal content at 29{degree sign}C be caused by an overestimation of mEPSP amplitudes? Please report the signal-to-noise ratio and plot mini amplitude distributions. Based on the signal-to-noise ratio, the authors should discuss this issue/ potentially correct for this.

4. Could the "non-perfect" homeostatic increase in release be due to limited GluR function/number rather than a limitation in homeostatic potentiation (Figure 1B, C)? For instance, whenever mEPSP amplitudes drop below {less than or equal to}0.35mV in GluRIIAm/r mutants homeostasis is disrupted/not perfect (Figure 1 and 6). Could this be simply due to too few receptors without affecting PHP? Please comment on these issues!

5. The terms "perfect and "imperfect" seem inappropriate, because "perfect" implies that the same EPSP amplitudes are maintained under the different experimental conditions. As shown in the figures (e.g. Figure 1C), this is of course not the case. The authors should come up with a different terminology here (e.g. "partial" vs. "full" EPSP recovery?)

6. Figure 4: Based on the fact that reversibility is the major finding of this study, it would be very informative to know more about the recovery time course. Is the EPSP recovery as rapid as the mEPSP recovery? The recovery of QC seems somewhat slower than the mEPSP recovery. This should be worked out in more detail. Could the authors fit the recovery time course with an exponential function to quantify a recovery time constant? The authors could then compare the time course of mEPSP and EPSP recovery (see also below). Even if the incorporation of new GluRs is comparably slow, the authors roughly know the time course (mEPSP recovery) and can relate it to the EPSP recovery.

The understanding of panel A would be greatly facilitated by color-coding the different experimental groups and by showing the averages of each group. Panels B, C: Could the authors include averages and fits? Moreover, it would be very helpful to add a panel showing EPSP amplitudes vs. experimental time. Plotting the relative recovery of mEPSP and EPSP amplitudes (plus fits) could visualize a potential delay in the reversibility of PHP.

7. Figure 5: The mEPSP amplitude decrease at 28.5{degree sign}C is rather small compared to 25{degree sign}C. Could "perfect homeostasis" be simply due to "imperfect" mEPSP decrease? This needs to be discussed!

8. Homeostasis block at 30{degree sign}C: Similar to point 4, homeostasis could be still expressed and the increase in QC could no longer be detected due to limited GluR number/function. If this possibility cannot be ruled out, the data would not support the conclusion that "signaling processes that maintain PHP in response to the dominant-negative transgene may break down at high temperatures" (line 325). The authors need to discuss this/ may need to change this conclusion.

9. Why do GluRIIAm/r mutants express PHP at 29{degree sign}C, but not at 30{degree sign}C?! As mentioned by the authors - do the synapses differ in size, morphology or GluR composition? Synapse morphology and GluR levels/composition needs to be investigated in GluRIIAm/r mutants at these two temperatures to get a better understanding of this phenotype.

10. Why do GluRIIAsp16 and GluRIIAm/r mutants display different phenotypes at 30{degree sign}C? When does GluRIIAsp16 "crash"? The authors should test GluRIIAsp16 mutants at higher temperatures until PHP fails. This would further support their conclusion that homeostasis is temperature sensitive at this synapse (as mentioned in the title). Moreover, investigation of NMJ morphology and GluR levels/ composition in GluRIIAsp16 and GluRIIAm/r mutants at 30{degree sign}C would be helpful.

MINOR POINTS

1. Some of the wording (crippled, weak EPSPs, crash etc.) should be changed.

2. Figure 3: In addition to the normalized data it would be helpful to show absolute data, because the authors report that parameters depend on developmental stage, temperature and perturbation.

3. In the discussion, the authors note "24 hours of halted dominant-negative expression provided no relief" (line 361). Does "relief" refer to PHP or the perturbation? mEPSP amplitudes did not recover during 24 hours, so one cannot draw any conclusions on the "lag" between PHP (EPSP/QC decrease) and the relief of the perturbation (mEPSP decrease) (see also above, 6.).

REVIEWER #2

This is a study investigating the reversibility and temperature sensitivity of homeostatic synaptic plasticity at Drosophila NMJ. Reversibility is a significant aspect of homeostatic plasticity that has been assumed by the field but has not been adequately demonstrated in vivo. Using temperature-sensitive genetic manipulations, the authors show nicely that presynaptic homeostatic potentiation (PHP) at fly NMJ is indeed reversible in a matter of days. Additionally, the authors investigated the effect of high temperature on PHP and found that PHP mechanism breaks down at high temperature. Overall, the work is interesting and the data of high quality. My main concern is related to the data interpretation of the PHP at high temperature experiments.

With MHC-Gal4 driver line, dominant negative GluIIA expression at 29 oC exhibited imperfect PHP, while with BG57-Gal4 driver line, the same genetic manipulation at 28.5 oC exhibited perfect PHP. It is unlikely that the 0.5 oC difference is solely responsible for the imperfect PHP. How does different driver lines affect PHP at high temperature? Is it due to different level of Gal4 expression?

I am puzzled by the data presented in figure 6. Table 1 shows that at 30 oC, both driver line control groups have reduced mEPSP amplitudes, nearly normal EPSP, and significantly increased QC in comparison to the WT line, suggesting some sort of homeostatic PHP mechanism is at play. However, in response to DN GluIIA expression, mEPSP, EPSP, and QC all drop further. Thus, my interpretation is that PHP mechanism is still relatively intact given how the NMJ maintained normal EPSP despite reduced mEPSP in the control lines. Instead, when mEPSP is reduced further by DN GluIIA expression, additional signaling processes are activated to reduce presynaptic release, or PHP mechanism is suppressed by DN GluIIA expression. I suggest replotting the data in Fig 6 by normalizing every parameter to the WT level instead of respective control driver lines, and carefully interpret the data based on the new plot.

Additionally, in both dominant negative GluRIIA lines (GluRIIA M/R driven by MHC- or BG57-Gal4 driver lines), PHP is almost completely gone, while in the GluRIIA deletion line, it is still robust albeit imperfect. Does this mean that different PHP mechanisms are engaged by these two types of perturbation, and the mechanism involved in the deletion line is less susceptible to high temperature? Why?

## References

[B1] Bergquist S, Dickman DK, Davis GW (2010) A hierarchy of cell intrinsic and target-derived homeostatic signaling. Neuron 66:220–234. 10.1016/j.neuron.2010.03.023 20434999 PMC2864777

[B2] Brusich DJ, Spring AM, Frank CA (2015) A single-cross, RNA interference-based genetic tool for examining the long-term maintenance of homeostatic plasticity. Front Cell Neurosci 9:107 10.3389/fncel.2015.00107 25859184 PMC4374470

[B3] Budnik V, Koh YH, Guan B, Hartmann B, Hough C, Woods D, Gorczyca M (1996) Regulation of synapse structure and function by the Drosophila tumor suppressor gene dlg. Neuron 17:627–640. 8893021 10.1016/s0896-6273(00)80196-8 PMC4661176

[B4] Budnik V, Zhong Y, Wu CF (1990) Morphological plasticity of motor axons in Drosophila mutants with altered excitability. J Neurosci 10:3754–3768. 1700086 10.1523/JNEUROSCI.10-11-03754.1990 PMC6570094

[B5] Burrone J, O’Byrne M, Murthy VN (2002) Multiple forms of synaptic plasticity triggered by selective suppression of activity in individual neurons. Nature 420:414–418. 10.1038/nature01242 12459783

[B6] Corrigan L, Redhai S, Leiblich A, Fan SJ, Perera SM, Patel R, Gandy C, Wainwright SM, Morris JF, Hamdy F, et al (2014) BMP-regulated exosomes from Drosophila male reproductive glands reprogram female behavior. J Cell Biol 206:671–688. 10.1083/jcb.201401072 25154396 PMC4151142

[B7] Cull-Candy SG, Miledi R, Trautmann A, Uchitel OD (1980) On the release of transmitter at normal, myasthenia gravis and myasthenic syndrome affected human end-plates. J Physiol 299:621–638. 10.1113/jphysiol.1980.sp013145 6103954 PMC1279245

[B8] Daniels RW, Collins CA, Chen K, Gelfand MV, Featherstone DE, DiAntonio A (2006) A single vesicular glutamate transporter is sufficient to fill a synaptic vesicle. Neuron 49:11–16. 10.1016/j.neuron.2005.11.032 16387635 PMC2248602

[B9] Daniels RW, Collins CA, Gelfand MV, Dant J, Brooks ES, Krantz DE, DiAntonio A (2004) Increased expression of the Drosophila vesicular glutamate transporter leads to excess glutamate release and a compensatory decrease in quantal content. J Neurosci 24:10466–10474. 10.1523/JNEUROSCI.3001-04.2004 15548661 PMC6730318

[B10] Davis GW, Bezprozvanny I (2001) Maintaining the stability of neural function: a homeostatic hypothesis. Annu Rev Physiol 63:847–869. 10.1146/annurev.physiol.63.1.847 11181978

[B11] Davis GW, DiAntonio A, Petersen SA, Goodman CS (1998) Postsynaptic PKA controls quantal size and reveals a retrograde signal that regulates presynaptic transmitter release in Drosophila. Neuron 20:305–315. 10.1016/S0896-6273(00)80458-4 9491991

[B12] Davis GW, Goodman CS (1998) Synapse-specific control of synaptic efficacy at the terminals of a single neuron. Nature 392:82–86. 10.1038/32176 9510251

[B13] Davis GW, Müller M (2015) Homeostatic control of presynaptic neurotransmitter release. Annu Rev Physiol 77:251–270. 10.1146/annurev-physiol-021014-071740 25386989

[B14] DiAntonio A, Petersen SA, Heckmann M, Goodman CS (1999) Glutamate receptor expression regulates quantal size and quantal content at the Drosophila neuromuscular junction. J Neurosci 19:3023–3032. 10191319 10.1523/JNEUROSCI.19-08-03023.1999 PMC6782296

[B15] Dickman DK, Davis GW (2009) The schizophrenia susceptibility gene dysbindin controls synaptic homeostasis. Science 326:1127–1130. 10.1126/science.1179685 19965435 PMC3063306

[B16] Dickman DK, Tong A, Davis GW (2012) Snapin is critical for presynaptic homeostatic plasticity. J Neurosci 32:8716–8724. 10.1523/JNEUROSCI.5465-11.2012 22723711 PMC3395587

[B17] Featherstone DE, Rushton E, Rohrbough J, Liebl F, Karr J, Sheng Q, Rodesch CK, Broadie K (2005) An essential Drosophila glutamate receptor subunit that functions in both central neuropil and neuromuscular junction. J Neurosci 25:3199–3208. 15788777 10.1523/JNEUROSCI.4201-04.2005 PMC2194804

[B18] Frank CA (2014) Homeostatic plasticity at the Drosophila neuromuscular junction. Neuropharmacology 78:63–74. 10.1016/j.neuropharm.2013.06.015 23806804 PMC3830618

[B19] Frank CA, Kennedy MJ, Goold CP, Marek KW, Davis GW (2006) Mechanisms underlying the rapid induction and sustained expression of synaptic homeostasis. Neuron 52:663–677. 10.1016/j.neuron.2006.09.029 17114050 PMC2673733

[B20] Frank CA, Pielage J, Davis GW (2009) A presynaptic homeostatic signaling system composed of the Eph receptor, ephexin, Cdc42, and CaV2.1 calcium channels. Neuron 61:556–569. 10.1016/j.neuron.2008.12.028 19249276 PMC2699049

[B21] Gaviño MA, Ford KJ, Archila S, Davis GW (2015) Homeostatic synaptic depression is achieved through a regulated decrease in presynaptic calcium channel abundance. eLife 4:e05473. 10.7554/eLife.05473 PMC4443758 25884248

[B22] Genç Ö, Dickman DK, Ma W, Tong A, Fetter RD, Davis GW (2017) MCTP is an ER-resident calcium sensor that stabilizes synaptic transmission and homeostatic plasticity. eLife 6:e22904. 10.7554/eLife.22904 PMC5449185 28485711

[B23] Goel A, Lee HK (2007) Persistence of experience-induced homeostatic synaptic plasticity through adulthood in superficial layers of mouse visual cortex. J Neurosci 27:6692–6700. 10.1523/JNEUROSCI.5038-06.2007 17581956 PMC2601561

[B24] Harris N, Braiser DJ, Dickman DK, Fetter RD, Tong A, Davis GW (2015) The innate immune receptor PGRP-LC controls presynaptic homeostatic plasticity. Neuron 88:1157–1164. 10.1016/j.neuron.2015.10.049 26687223 PMC4718712

[B25] Hazelrigg T, Levis R, Rubin GM (1984) Transformation of white locus DNA in Drosophila: dosage compensation, zeste interaction, and position effects. Cell 36:469–481. 10.1016/0092-8674(84)90240-X 6319027

[B26] Kauwe G, Tsurudome K, Penney J, Mori M, Gray L, Calderon MR, Elazouzzi F, Chicoine N, Sonenberg N, Haghighi AP (2016) Acute fasting regulates retrograde synaptic enhancement through a 4E-BP-dependent mechanism. Neuron 92:1204–1212. 10.1016/j.neuron.2016.10.063 27916456 PMC5797711

[B27] Kikuma K, Li X, Kim D, Sutter D, Dickman DK (2017) Extended synaptotagmin localizes to presynaptic ER and promotes neurotransmission and synaptic growth in Drosophila. Genetics 207:993–1006. 28882990 10.1534/genetics.117.300261 PMC5676231

[B28] Kim YJ, Bao H, Bonanno L, Zhang B, Serpe M (2012) Drosophila Neto is essential for clustering glutamate receptors at the neuromuscular junction. Genes Dev 26:974–987. 10.1101/gad.185165.111 22499592 PMC3347794

[B29] Kim YJ, Igiesuorobo O, Ramos CI, Bao H, Zhang B, Serpe M (2015) Prodomain removal enables neto to stabilize glutamate receptors at the Drosophila neuromuscular junction. PLoS Genetics 11:e1004988. 10.1371/journal.pgen.1004988 25723514 PMC4344203

[B30] Kiragasi B, Wondolowski J, Li Y, Dickman DK (2017) A presynaptic glutamate receptor subunit confers robustness to neurotransmission and homeostatic potentiation. Cell Reports 19:2694–2706. 10.1016/j.celrep.2017.06.003 28658618 PMC5538789

[B31] Lee J, Wu CF (2010) Orchestration of stepwise synaptic growth by K+ and Ca2+ channels in Drosophila. J Neurosci 30:15821–15833. 10.1523/JNEUROSCI.3448-10.2010 21106821 PMC3075884

[B32] Lnenicka GA, Mellon D Jr. (1983a) Changes in electrical properties and quantal current during growth of identified muscle fibres in the crayfish. J Physiol 345:261–284. 6663501 10.1113/jphysiol.1983.sp014977 PMC1193796

[B33] Lnenicka GA, Mellon D Jr. (1983b) Transmitter release during normal and altered growth of identified muscle fibres in the crayfish. J Physiol 345:285–296. 6141287 10.1113/jphysiol.1983.sp014978 PMC1193797

[B34] Marder E, Bucher D (2007) Understanding circuit dynamics using the stomatogastric nervous system of lobsters and crabs. Annu Rev Physiol 69:291–316. 10.1146/annurev.physiol.69.031905.161516 17009928

[B35] Marder E, Goaillard JM (2006) Variability, compensation and homeostasis in neuron and network function. Nat Rev Neurosci 7:563–574. 10.1038/nrn1949 16791145

[B36] Marder E, Haddad SA, Goeritz ML, Rosenbaum P, Kispersky T (2015) How can motor systems retain performance over a wide temperature range? Lessons from the crustacean stomatogastric nervous system. J Comp Physiol A Neuroethol Sens Neural Behav Physiol Neuroethol Sens Neural Behav Physiol 201:851–856. 10.1007/s00359-014-0975-2 PMC4552768 25552317

[B37] Marder E, Prinz AA (2002) Modeling stability in neuron and network function: the role of activity in homeostasis. Bioessays 24:1145–1154. 10.1002/bies.10185 12447979

[B38] Marie B, Pym E, Bergquist S, Davis GW (2010) Synaptic homeostasis is consolidated by the cell fate gene gooseberry, a Drosophila pax3/7 homolog. J Neurosci 30:8071–8082. 10.1523/JNEUROSCI.5467-09.2010 20554858 PMC3291498

[B39] McGuire SE, Le PT, Osborn AJ, Matsumoto K, Davis RL (2003) Spatiotemporal rescue of memory dysfunction in Drosophila. Science 302:1765–1768. 10.1126/science.1089035 14657498

[B40] Müller M, Davis GW (2012) Transsynaptic control of presynaptic Ca(2)(+) influx achieves homeostatic potentiation of neurotransmitter release. Curr Biol 22:1102–1108. 10.1016/j.cub.2012.04.018 22633807 PMC4367479

[B41] Müller M, Genç Ö, Davis GW (2015) RIM-binding protein links synaptic homeostasis to the stabilization and replenishment of high release probability vesicles. Neuron 85:1056–1069. 10.1016/j.neuron.2015.01.024 25704950 PMC4354699

[B42] Müller M, Liu KS, Sigrist SJ, Davis GW (2012) RIM controls homeostatic plasticity through modulation of the readily-releasable vesicle pool. J Neurosci 32:16574–16585. 10.1523/JNEUROSCI.0981-12.2012 23175813 PMC3523185

[B43] Müller M, Pym EC, Tong A, Davis GW (2011) Rab3-GAP controls the progression of synaptic homeostasis at a late stage of vesicle release. Neuron 69:749–762. 10.1016/j.neuron.2011.01.025 21338884 PMC3059509

[B44] Murthy VN, Schikorski T, Stevens CF, Zhu Y (2001) Inactivity produces increases in neurotransmitter release and synapse size. Neuron 32:673–682. 11719207 10.1016/s0896-6273(01)00500-1

[B45] O’Brien RJ, Kamboj S, Ehlers MD, Rosen KR, Fischbach GD, Huganir RL (1998) Activity-dependent modulation of synaptic AMPA receptor accumulation. Neuron 21:1067–1078. 9856462 10.1016/s0896-6273(00)80624-8

[B46] Orr BO, Fetter RD, Davis GW (2017a) Retrograde semaphorin-plexin signalling drives homeostatic synaptic plasticity. Nature 550:109–113. 28953869 10.1038/nature24017 PMC5907800

[B47] Orr BO, Gorczyca D, Younger MA, Jan LY, Jan YN, Davis GW (2017b) Composition and control of a Deg/ENaC channel during presynaptic homeostatic plasticity. Cell Reports 20:1855–1866. 28834749 10.1016/j.celrep.2017.07.074 PMC5599149

[B48] Parrish JZ, Kim CC, Tang L, Bergquist S, Wang T, Derisi JL, Jan LY, Jan YN, Davis GW (2014) Kruppel mediates the selective rebalancing of ion channel expression. Neuron 82:537–544. 10.1016/j.neuron.2014.03.015 24811378 PMC4104505

[B49] Penney J, Tsurudome K, Liao EH, Elazzouzi F, Livingstone M, Gonzalez M, Sonenberg N, Haghighi AP (2012) TOR is required for the retrograde regulation of synaptic homeostasis at the Drosophila neuromuscular junction. Neuron 74:166–178. 10.1016/j.neuron.2012.01.030 22500638

[B50] Petersen SA, Fetter RD, Noordermeer JN, Goodman CS, DiAntonio A (1997) Genetic analysis of glutamate receptors in Drosophila reveals a retrograde signal regulating presynaptic transmitter release. Neuron 19:1237–1248. 10.1016/S0896-6273(00)80415-8 9427247

[B51] Plomp JJ, Van Kempen GT, De Baets MB, Graus YM, Kuks JB, Molenaar PC (1995) Acetylcholine release in myasthenia gravis: regulation at single end-plate level. Ann Neurol 37:627–636. 10.1002/ana.410370513 7755358

[B52] Plomp JJ, van Kempen GT, Molenaar PC (1992) Adaptation of quantal content to decreased postsynaptic sensitivity at single endplates in alpha-bungarotoxin-treated rats. J Physiol 458:487–499. 10.1113/jphysiol.1992.sp019429 1302275 PMC1175167

[B53] Ramos CI, Igiesuorobo O, Wang Q, Serpe M (2015) Neto-mediated intracellular interactions shape postsynaptic composition at the Drosophila neuromuscular junction. PLoS Genetics 11:e1005191 10.1371/journal.pgen.1005191 25905467 PMC4408064

[B54] Rasse TM, Fouquet W, Schmid A, Kittel RJ, Mertel S, Sigrist CB, Schmidt M, Guzman A, Merino C, Qin G, et al (2005) Glutamate receptor dynamics organizing synapse formation in vivo. Nat Neurosci 8:898–905. 10.1038/nn1484 16136672

[B55] Redhai S, Hellberg JE, Wainwright M, Perera SW, Castellanos F, Kroeger B, Gandy C, Leiblich A, Corrigan L, Hilton T, et al (2016) Regulation of dense-core granule replenishment by autocrine BMP signalling in Drosophila secondary cells. PLoS Genet 12:e1006366. 10.1371/journal.pgen.1006366 27727275 PMC5065122

[B56] Rinberg A, Taylor AL, Marder E (2013) The effects of temperature on the stability of a neuronal oscillator. PLoS Comput Biol 9:e1002857. 10.1371/journal.pcbi.1002857 23326223 PMC3542102

[B57] Rongo C, Kaplan JM (1999) CaMKII regulates the density of central glutamatergic synapses in vivo. Nature 402:195–199. 10.1038/46065 10647013

[B58] Rutherford LC, DeWan A, Lauer HM, Turrigiano GG (1997) Brain-derived neurotrophic factor mediates the activity-dependent regulation of inhibition in neocortical cultures. J Neurosci 17:4527–4535. 9169513 10.1523/JNEUROSCI.17-12-04527.1997 PMC6573348

[B59] Schmid A, Hallermann S, Kittel RJ, Khorramshahi O, Frölich AM, Quentin C, Rasse TM, Mertel S, Heckmann M, Sigrist SJ (2008) Activity-dependent site-specific changes of glutamate receptor composition in vivo. Nat Neurosci 11:659–666. 10.1038/nn.2122 18469810

[B60] Schmid A, Qin G, Wichmann C, Kittel RJ, Mertel S, Fouquet W, Schmidt M, Heckmann M, Sigrist SJ (2006) Non-NMDA-type glutamate receptors are essential for maturation but not for initial assembly of synapses at Drosophila neuromuscular junctions. J Neurosci 26:11267–11277. 10.1523/JNEUROSCI.2722-06.2006 17079654 PMC6674544

[B61] Schuster CM, Davis GW, Fetter RD, Goodman CS (1996a) Genetic dissection of structural and functional components of synaptic plasticity. I. Fasciclin II controls synaptic stabilization and growth. Neuron 17:641–654. 8893022 10.1016/s0896-6273(00)80197-x

[B62] Schuster CM, Davis GW, Fetter RD, Goodman CS (1996b) Genetic dissection of structural and functional components of synaptic plasticity. II. Fasciclin II controls presynaptic structural plasticity. Neuron 17:655–667. 8893023 10.1016/s0896-6273(00)80198-1

[B63] Sigrist SJ, Reiff DF, Thiel PR, Steinert JR, Schuster CM (2003) Experience-dependent strengthening of Drosophila neuromuscular junctions. J Neurosci 23:6546–6556. 12878696 10.1523/JNEUROSCI.23-16-06546.2003 PMC6740647

[B64] Spring AM, Brusich DJ, Frank CA (2016) C-terminal Src kinase gates homeostatic synaptic plasticity and regulates fasciclin II expression at the Drosophila neuromuscular junction. PLoS Genet 12:e1005886. 10.1371/journal.pgen.1005886 26901416 PMC4764653

[B65] Staley BK, Irvine KD (2010) Warts and Yorkie mediate intestinal regeneration by influencing stem cell proliferation. Curr Biol 20:1580–1587. 10.1016/j.cub.2010.07.041 20727758 PMC2955330

[B66] Swanwick CC, Murthy NR, Kapur J (2006) Activity-dependent scaling of GABAergic synapse strength is regulated by brain-derived neurotrophic factor. Mol Cell Neurosci 31:481–492. 10.1016/j.mcn.2005.11.002 16330218 PMC2842119

[B67] Tang LS, Taylor AL, Rinberg A, Marder E (2012) Robustness of a rhythmic circuit to short- and long-term temperature changes. J Neurosci 32:10075–10085. 10.1523/JNEUROSCI.1443-12.2012 22815521 PMC3491657

[B68] Thiagarajan TC, Lindskog M, Tsien RW (2005) Adaptation to synaptic inactivity in hippocampal neurons. Neuron 47:725–737. 10.1016/j.neuron.2005.06.037 16129401

[B69] Turrigiano GG (2008) The self-tuning neuron: synaptic scaling of excitatory synapses. Cell 135:422–435. 10.1016/j.cell.2008.10.008 18984155 PMC2834419

[B70] Turrigiano GG (2017) The dialectic of Hebb and homeostasis. Philos Trans R Soc Lond B Biol Sci 5:372. 10.1098/rstb.2016.0258 PMC5247594 28093556

[B71] Turrigiano GG, Leslie KR, Desai NS, Rutherford LC, Nelson SB (1998) Activity-dependent scaling of quantal amplitude in neocortical neurons. Nature 391:892–896. 10.1038/36103 9495341

[B72] Ueda A, Wu CF (2015) The role of cAMP in synaptic homeostasis in response to environmental temperature challenges and hyperexcitability mutations. Front Cell Neurosci 9:10. 10.3389/fncel.2015.00010 25698925 PMC4313691

[B73] Wang HL, Zhang Z, Hintze M, Chen L (2011) Decrease in calcium concentration triggers neuronal retinoic acid synthesis during homeostatic synaptic plasticity. J Neurosci 31:17764–17771. 10.1523/JNEUROSCI.3964-11.2011 22159093 PMC3457695

[B74] Wang T, Hauswirth AG, Tong A, Dickman DK, Davis GW (2014) Endostatin is a trans-synaptic signal for homeostatic synaptic plasticity. Neuron 83:616–629. 10.1016/j.neuron.2014.07.003 25066085 PMC4133507

[B75] Wang T, Jones RT, Whippen JM, Davis GW (2016a) alpha2delta-3 is required for rapid transsynaptic homeostatic signaling. Cell Reports 16:2875–2888. 27626659 10.1016/j.celrep.2016.08.030 PMC5599162

[B76] Wang X, Pinter MJ, Rich MM (2016b) Reversible recruitment of a homeostatic reserve pool of synaptic vesicles underlies rapid homeostatic plasticity of quantal content. J Neurosci 36:828–836. 26791213 10.1523/JNEUROSCI.3786-15.2016 PMC4719018

[B77] Wefelmeyer W, Puhl CJ, Burrone J (2016) Homeostatic plasticity of subcellular neuronal structures: from inputs to outputs. Trends Neurosci 39:656–667. 10.1016/j.tins.2016.08.004 27637565 PMC5236059

[B78] Weyhersmüller A, Hallermann S, Wagner N, Eilers J (2011) Rapid active zone remodeling during synaptic plasticity. J Neurosci 31:6041–6052. 10.1523/JNEUROSCI.6698-10.2011 21508229 PMC6632979

[B79] Whiting B, Moiseff A, Rubio ME (2009) Cochlear nucleus neurons redistribute synaptic AMPA and glycine receptors in response to monaural conductive hearing loss. Neuroscience 163:1264–1276. 10.1016/j.neuroscience.2009.07.049 19646510 PMC2760652

[B80] Wierenga CJ, Ibata K, Turrigiano GG (2005) Postsynaptic expression of homeostatic plasticity at neocortical synapses. J Neurosci 25:2895–2905. 10.1523/JNEUROSCI.5217-04.2005 15772349 PMC6725152

[B81] Younger MA, Müller M, Tong A, Pym EC, Davis GW (2013) A presynaptic ENaC channel drives homeostatic plasticity. Neuron 79:1183–1196. 10.1016/j.neuron.2013.06.048 23973209 PMC3784986

[B82] Zhong Y, Budnik V, Wu CF (1992) Synaptic plasticity in Drosophila memory and hyperexcitable mutants: role of cAMP cascade. J Neurosci 12:644–651. 1371316 10.1523/JNEUROSCI.12-02-00644.1992 PMC6575602

[B83] Zhong Y, Wu CF (2004) Neuronal activity and adenylyl cyclase in environment-dependent plasticity of axonal outgrowth in Drosophila. J Neurosci 24:1439–1445. 10.1523/JNEUROSCI.0740-02.2004 14960616 PMC1289273

